# Outlier analyses and genome-wide association study identify *glgC* and *ERD6-like 4* as candidate genes for foliar water-soluble carbohydrate accumulation in *Trifolium repens*

**DOI:** 10.3389/fpls.2022.1095359

**Published:** 2023-01-09

**Authors:** Sofie M. Pearson, Andrew G. Griffiths, Paul Maclean, Anna C. Larking, S. Won Hong, Ruy Jauregui, Poppy Miller, Catherine M. McKenzie, Peter J. Lockhart, Jennifer A. Tate, John L. Ford, Marty J. Faville

**Affiliations:** ^1^ School of Natural Sciences, Massey University, Palmerston North, New Zealand; ^2^ Resilient Agriculture, AgResearch Grasslands, Palmerston North, New Zealand; ^3^ Grasslands, PGG Wrightson Seeds Limited, Palmerston North, New Zealand

**Keywords:** genome-wide association study, genotyping-by-sequencing, outlier detection, white clover, water-soluble carbohydrate

## Abstract

Increasing water-soluble carbohydrate (WSC) content in white clover is important for improving nutritional quality and reducing environmental impacts from pastoral agriculture. Elucidation of genes responsible for foliar WSC variation would enhance genetic improvement by enabling molecular breeding approaches. The aim of the present study was to identify single nucleotide polymorphisms (SNPs) associated with variation in foliar WSC in white clover. A set of 935 white clover individuals, randomly sampled from five breeding pools selectively bred for divergent (low or high) WSC content, were assessed with 14,743 genotyping-by-sequencing SNPs, using three outlier detection methods: PCAdapt, BayeScan and KGD-F_ST_. These analyses identified 33 SNPs as discriminating between high and low WSC populations and putatively under selection. One SNP was located in the intron of *ERD6-like 4*, a gene coding for a sugar transporter located on the vacuole membrane. A genome-wide association study using a subset of 605 white clover individuals and 5,757 SNPs, identified a further 12 SNPs, one of which was associated with a starch biosynthesis gene, glucose-1-phosphate adenylyltransferase, *glgC*. Our results provide insight into genomic regions underlying WSC accumulation in white clover, identify candidate genomic regions for further functional validation studies, and reveal valuable information for marker-assisted or genomic selection in white clover.

## 1 Introduction

White clover (*Trifolium repens* L.) is sown in temperate pastures globally, as it provides high quality forage for ruminants and is a source of bioavailable nitrogen, fixed through symbiosis with soil *Rhizobium* bacteria (Ulyatt, 1997). It is a recent (15 – 28,000 years ago) allotetraploid that resulted from the hybridization of two diploid *Trifolium* species, *T. occidentale* and *T. pallescens* (Ellison et al., 2006; Griffiths et al., 2019). Retention of the combined genomes as *T. occidentale* and *T. pallescens*-derived subgenomes likely underpins the broad adaptation and phenotypic plasticity of this agronomically successful species (Griffiths et al., 2019). White clover foliage has a high concentration of crude protein but a relatively low concentration of water-soluble carbohydrate (WSC) (Cosgrove et al., 2009). Foliar WSC is important because it provides readily available energy to the rumen microbiome, which improves the efficiency of protein utilisation by the animal (Woodfield et al., 2001). Higher levels of WSC available for consumption by ruminant microbes enables a shift in the partitioning of digested nitrogen, with less excreted as urea and more utilised for animal growth and production (Easton et al., 2009). Breeding for increased foliar WSC in pasture species, including white clover, can therefore have beneficial effects for the environment as less nitrogen is lost *via* urine and dung to nitrous oxide emission and nitrate leaching (Luo et al., 2015; Selbie et al., 2015). In addition to improved nutritional quality and positive environmental outcomes, WSC has been found to be important in conferring cold tolerance and drought resistance in plants due to its role in osmotic adjustment (Kerepesi and Galiba, 2000; Dalmannsdóttir et al., 2001; Livingston et al., 2009).

Several aspects of WSC composition and variation in white clover plants have been studied, including seasonal and diurnal foliar WSC variation (Michell, 1973; Ruckle et al., 2018; Kagan et al., 2020). While research has addressed the genetic control of WSC accumulation in stolons (Inostroza et al., 2018), little is known about the genetic mechanisms underlying foliar WSC accumulation in white clover. Improved understanding of the genes that influence foliar WSC accumulation would support the development and application of molecular breeding tools, such as marker-assisted and genomic selection, that could be used by breeders to accelerate genetic improvement of this trait.

Two complementary approaches may be used to detect genomic loci linked to trait phenotype variation, as a means to identify candidate genes. Outlier analysis can be used to identify single nucleotide polymorphisms (SNPs) that differentiate populations with divergent phenotypes. This approach most commonly involves F_ST_-based tests (Antao et al., 2008; Whitlock and Lotterhos, 2015) or principal component analyses (PCA) (Luu et al., 2017) to identify differentiating loci that are distinct from those under neutral selection. Genome-wide association studies (GWAS) offer a second approach, utilising SNP markers across the entire genome with the goal of associating specific variants with phenotypic variation, as measured in a population or a panel of diverse individuals. This method can be used in both model and non-model organisms and has successfully identified genes underlying traits in forage species (Arojju et al., 2016; Sakiroglu and Brummer, 2017; Biazzi et al., 2017). Both outlier approaches and GWAS require a preliminary assessment of population structure to avoid false positive associations (Sul et al., 2018).

Selective breeding to create experimental white clover populations with divergent levels of foliar WSC was previously undertaken in two New Zealand breeding programmes, with five discrete breeding pools. Three were described by Widdup et al. (2010) and conducted over four cycles of divergent recurrent selection, and the remaining two pools were part of another programme (Mr. John Ford, pers. comm.) in which selection took place over six cycles. Both programmes included pools in different leaf size classes (large leaf and small leaf). The divergently-selected populations in these pools represent a valuable genetic resource for investigating the genetic basis of WSC accumulation, including the relationship between leaf size and WSC levels (Woodfield et al., 2001).

Genotyping-by-sequencing (GBS), enables highly efficient and cost-effective simultaneous SNP discovery and genotyping (Elshire et al., 2011; Poland et al., 2012b) and has been implemented in numerous forage species (Biazzi et al., 2017; Sakiroglu and Brummer, 2017; Faville et al., 2018; Guo et al., 2018), including white clover (Wright et al., 2017; Griffiths et al., 2019). In our study, we applied GBS in the five white clover pools to support investigation of genomic regions and loci under selection for WSC accumulation. Analyses were based on genome-wide GBS-derived SNP data from individuals within the breeding pools, targeting three generational time points within each pool. The overall aim was to identify SNPs associated with foliar WSC accumulation, that may subsequently be developed and used to support gene discovery and molecular breeding approaches in white clover populations to aid breeding for increased WSC accumulation in white clover.

There were three principal objectives: (1) to confirm that foliar WSC phenotypes were significantly and directionally different amongst the populations used in the study, ensuring that subsequent genetic studies were performed on truly divergent phenotypes; (2) to establish whether selective breeding had altered WSC independently of changing leaf area in those populations; and (3) apply outlier detection and GWAS approaches to identify SNPs associated with foliar WSC accumulation.

## 2 Materials and methods

### 2.1 Plant material

**Figure 1:**
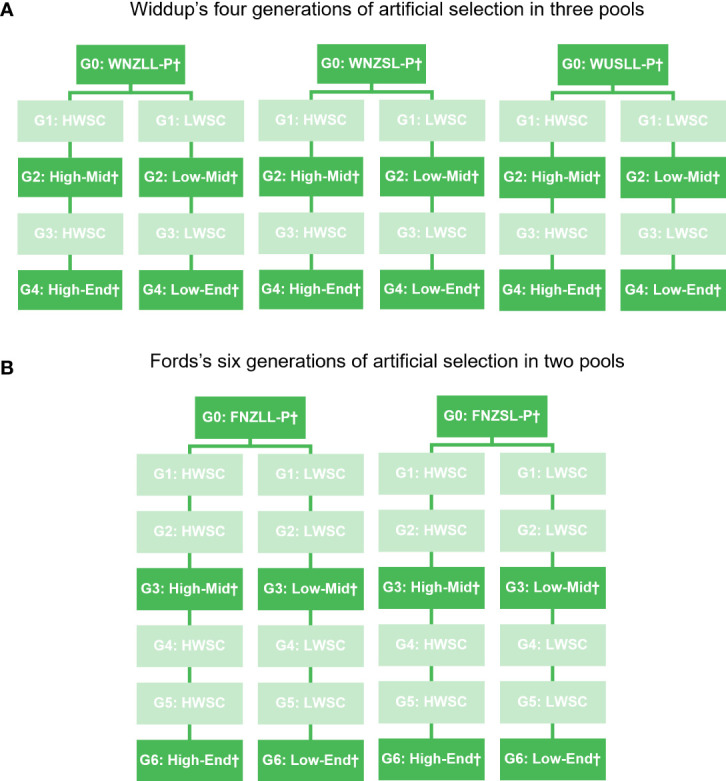
Schematic representation of white clover populations from the **(A)** Widdup et al. (2010) and **(B)** Ford breeding programmes. G, generation; W, Widdup; F, Ford; NZ, New Zealand/Aotearoa; US, United States of America; LL, large leaf; SL, small leaf; P, Parent generation; HWSC and High, high water-soluble carbohydrate (WSC); LWSC and Low, low WSC; Mid, Middle generation; and End, End generation. † denotes populations used in phenotyping and genotyping studies.

The plant material used in this study was bred and supplied by Grasslands Innovation Ltd. Selective breeding for foliar levels of water-soluble carbohydrate (WSC) in white clover was completed previously in two breeding programmes, one consisting of four cycles of recurrent selection in three breeding pools (Widdup et al., 2010) and the other over six cycles in two pools (Mr John Ford, pers. comm.) (**Figure 1**). In all breeding pools, divergent selection was undertaken at each cycle to create populations with either low or high levels of foliar WSC, so that at each generation there is both a low and a high WSC population. In the programme described by Widdup et al. (2010) there were 24 populations generated (3 breeding pools × 4 cycles × low/high WSC) and in the Ford programme there were also 24 populations (2 breeding pools × 6 cycles × low/high WSC). Adding the five parental generations, a total of 53 populations were available for evaluation. Of these, 25 were chosen for phenotyping and genotypic analyses (**Figure 1**). These were the parental, middle and end generation populations within each pool. The middle generation was cycle 2 or cycle 3 and the end generation cycle 4 or 6, for the Widdup and Ford pools, respectively. Seed from all populations was acquired from the Margot Forde Germplasm Centre (Palmerston North, New Zealand).

Nomenclature for population names is: W = Widdup, F = Ford; NZ = New Zealand, US = United States of America; LL = large leaf, SL = small leaf; Low = low water-soluble carbohydrate (WSC), High = high WSC; P = parental generation, Mid = middle generation and End = end generation. The 25 populations were: WNZLL-Low-End, WNZLL-Low-Mid, WNZLL-Parent, WNZLL-High-Mid, WNZLL-High-End, WNZSL-Low-End, WNZSL-Low-Mid, WNZSL-Parent, WNZSL-High-Mid, WNZSL-High-End, WUSLL-Low-End, WUSLL-Low-Mid, WUSLL-Parent, WUSLL-High-Mid, WUSLL-High-End, FNZLL-Low-End, FNZLL-Low-Mid, FNZLL-Parent, FNZLL-High-Mid, FNZLL-High-End, FNZSL-Low-End, FNZSL-Low-Mid, FNZSL-Parent, FNZSL-High-Mid, and FNZSL-High-End.

### 2.2 Population establishment and experimental design

Approximately 100 seeds from each of the 25 populations were germinated, planted into propagation trays and grown under standard greenhouse conditions for two months. A total of 900 plants from the 25 populations (180 plants per breeding pool) were then randomly selected and transplanted into 2L pots for phenotyping. In each breeding pool, 60 plants per parent population (Parent) were randomly selected, along with 30 plants each from the middle generation low and high WSC populations (Low-Mid, High-Mid) and 30 plants each from the end generation low and high WSC populations (Low-End, High-End). Potted plants were kept in the greenhouse to establish for two weeks before being placed outside at Palmerston North, New Zealand (40.38°S, 175.61°E), in autumn, late May 2017, in a randomised Latin square design with three replicate blocks. The block was set up on a 9 × 9 m concrete pad and plants were set at 30 cm centre-to-centre spacing.

### 2.3 Plant phenotyping

#### 2.3.1 Water-soluble carbohydrate phenotyping using near infra-red reflectance spectroscopy

Leaves from the 900 plants were sampled over three consecutive days in November 2017. One replicate block was harvested per day, between 8:00 and 10:00 am to minimise diurnal variation in WSC levels and to be consistent with the methodology originally used for breeding these divergent WSC selections (Widdup et al., 2010). Thirty fully expanded, healthy leaf laminae were removed per plant to constitute a single plant sample, snap frozen in liquid nitrogen, and stored at -20°C before being freeze-dried and milled. Samples from two of the three replicate blocks (*n* = 600) were analysed by near infra-red spectroscopy (NIRS) at the Massey University Nutrition Laboratory, (Palmerston North, New Zealand) for nutritive quality attributes including WSC concentration (g kg^-1^ dry matter). Two NIRS calibrations were used initially to determine WSC: (a) total soluble sugars and starch (SSS) (Corson et al., 1999), the calibration originally used in the Widdup et al. (2010) and Ford breeding programmes; (b) WSC-NIRS; the sum of high molecular weight (HMW)- and low molecular weight (LMW)-WSC fractions estimated using a calibration developed from perennial ryegrass (Cosgrove et al., 2009).

#### 2.3.2 Leaf area phenotyping

Leaf area was assessed for each of the five pools. A total of 450 of the 900 plants were sampled from every second column, across all three blocks (150 plants per block). Four leaves per plant (first leaves from the stolon tip with fully opened laminae) were collected, glued to 1 mm graph paper and scanned. Scanned images were converted to binary in ImageJ (Schindelin et al., 2012), a global calibration curve was set to convert from pixel distance to actual distance (cm), and leaf area (cm^2^) was estimated. The mean of four leaves was used as a proxy for average plant leaf area.

#### 2.3.3 Phenotype statistical analyses and correlation investigation

Estimated Marginal Means for the WSC phenotype, using SSS, and leaf area were calculated for each population using a linear mixed model in the R package “*emmeans*” v 1.4.2 (Lenth et al., 2020) that accounted for treatment and spatial variation (**Equation 1**).


y~ TrtC + (1 | Block) + (1 | Block:Column) + (1 | Block:Row)

Where: *y* is the phenotypic trait, and *TrtC* is the interaction between Pool, Generation (i.e., Parent, Mid and End) and *Trt* (i.e., None (Parent; no selection), high WSC and low WSC).

Leaf area data were transformed by square root such that residuals conformed to constant variance and normality (**Supplementary Figure 1**). SSS required no transformation as residual assumptions were met. Leaf area data were back-transformed to derive population-fitted values on the original cm^2^ scale. The Parent population was used as the baseline for comparisons between populations within each pool. Significant differences in these comparisons were investigated using “*emmeans*” at α = 0.05. The Low-End population was then used as the baseline for the SSS dataset so differences between the Low-End and High-End populations for each pool could be determined.

Three hundred plants common to the sets used for evaluation of WSC and leaf area were used for phenotypic correlation analysis (Pearson correlation). This dataset was then split further into five datasets corresponding to the five pools (WNZLL, WNZSL, WUSLL, FNZLL and FNZSL) within which correlations between the two variables were investigated. Prior to correlation analysis, leaf area and SSS data were evaluated for normality using the Shapiro-Wilk Normality Test implemented in R (Royston, 1995; R Core Team, 2019). Data that did not follow a normal distribution were transformed using Box-Cox Transformation analysis (Venables and Ripley, 2002). Models were created for each pool using R statistical software, and adjusted coefficient of determination (r^2^) values of the lines of best fit were used to initially compare the predictive abilities of leaf area for SSS. Regression analysis amongst populations was then conducted using linear mixed models constructed for each pool. Models were constructed with SSS as the dependent variable and the interaction between leaf area and population was used as the independent variable. The Parent population for each pool was used as the baseline for comparison. Adjusted r^2^ was reported for both types of linear models (SSS ~ leaf area and SSS ~ 0 + Population * leaf area) to avoid false inflation of r^2^ values. Residuals were checked for normality and assumptions were met; thus, no data transformation was required. The R scripts used to produce estimated phenotype means and perform correlation and regression analyses can be found at https://github.com/SofiePearson/White_Clover_WSC_Outlier_Detection_GWAS.

### 2.4 Plant genotyping

#### 2.4.1 Genotyping-by-sequencing library preparation and sequencing

Genomic DNA was extracted from 1,536 white clover individuals (approximately 60 individuals from each of the 25 populations) using the freeze-dried tissue protocol described in Anderson et al. (2018). A subset of 1,175 individuals, comprising 47 from each of the 25 populations, were chosen for genotyping-by-sequencing (GBS). Each GBS library consisted of 94 samples, plus one negative control (water) and one positive control DNA from an inbred white clover, “S9” (Griffiths et al., 2019). Thirteen GBS libraries were created in total, and any spare wells filled with duplicated DNA chosen at random from all populations, including duplication of samples within a library and duplication of samples among libraries. GBS libraries were constructed following Poland et al. (2012b) using restriction enzymes *PstI* and *MspI*, with some modifications (see Supplementary Methods). Each library was then sequenced in parallel on two lanes of a flow cell on an Illumina HiSeq 2500 (Illumina, San Diego, CA, USA) at Invermay Agricultural Centre (AgResearch, Mosgiel, New Zealand).

#### 2.4.2 SNP calling, filtering and genotyping-by-sequencing library quality control

Raw data FASTQ files containing sequence reads were processed for SNP identification using Trait Analysis by aSSociation, Evolution and Linkage (TASSEL) v 5.0 (Glaubitz et al., 2014), using default parameters except minor allele frequency was set to 0.01. An AgResearch white clover genome assembly (version 5) was used as the reference genome (Griffiths et al., 2019). Raw sequence data from 1,222 samples, 13 positive controls and 13 negative samples were analysed together. Sequence reads were first trimmed to 64 bp and identical reads were grouped into sequence tags. The sequence tags were then aligned to the reference genome using Burrows-Wheeler Alignment (BWA) tool (Li and Durbin, 2009).

After SNP calling, all filtering was performed using VCFtools v 0.1.16 (Danecek et al., 2011). To provide a SNP marker dataset for downstream analyses, the marker set was restricted to high quality SNPs: biallelic SNPs with read depth range of 5 – 150, missing genotype data ≤ 20% per SNP, and minor allele frequency ≥ 0.03. Samples with a large proportion of missing data were removed from the dataset and negative control samples were removed after checking that they did not contain unduly high levels of sequence data.

### 2.5 Analysis of population genetic structure and variation

Genetic structure in the dataset was explored by Discriminant Analysis of Principal Components (DAPC) (Jombart et al., 2010), implemented in “*adegenet*” v 2.1.1 (Jombart, 2008) for R software v 3.6.1 (R Core Team, 2019). The analysis used 14,743 SNPs from 1,113 individuals in 24 populations (WNZSL-Parent population excluded due to high sample missing data). *K-*means clustering was used to detect the number of clusters amongst the samples, without prior assumptions of assignment based on pool or population. *K*-means was run sequentially from 1 to 40 genetic clusters (*K*), with 800 principal components retained, accounting for approximately 90% of the total genetic variation. The optimal clustering solution corresponded with the lowest Bayesian Information Criterion (BIC). Individual assignment from the *a priori* grouping to the *K*-means determined clusters was visualised and compared using the R package “*ade4*” v 1.7-15 (Chessel et al., 2004).

DAPC analysis was implemented using the cross-validation “*xvalDapc()*” function, and was run with 100 replicates from 1 – 50 principal components (PCs) with individuals grouped based on the *K*-means determined number of clusters. This cross-validation method determined 6 PCs should be used, hence the final “*dacp()*” was run with “n.pca = 6” and 6 discriminant functions (DFs) retained. A scatter plot of individuals grouped by *K*-means on DFs was created using “*adegenet*” (Jombart, 2008).

Genetic variation within and among populations was assessed by Analysis of Molecular Variance (AMOVA) implemented with “*poppr*” v 2.9.1 (Kamvar et al., 2014) in R. This was conducted for all 24 populations, as well as among genetic clusters identified using the most supported DAPC *K*-value (*K* = 11) with 9,999 permutations (Excoffier et al., 1992; Kamvar et al., 2014). A matrix of pairwise genetic differentiation between all population pairs from two possible population structures (the *a priori* of *K* = 24 and the DAPC-determined grouping of *K* = 11) was also computed using the R package “*hierfstat*” v 0.04-22 (Goudet and Jombart, 2015) using the fixation index F_ST_ (Weir and Cockerham, 1984). The R scripts used to perform population genetic structure analyses can be found at https://github.com/SofiePearson/White_Clover_WSC_Outlier_Detection_GWAS.

### 2.6 Detection of loci under selection

Three approaches were used to analyse the 14,743 SNP dataset for loci under divergent selection: PCAdapt (Luu et al., 2017), BayeScan (Foll and Gaggiotti, 2008) and an F_ST_ outlier detection approach in the package Kinship using Genotyping-by-sequencing with Depth adjustment (KGD, available from https://github.com/AgResearch/KGD.git) (Dodds et al., 2015). Missing data for SNPs were not imputed.

#### 2.6.1 PCAdapt

Individuals from the DAPC analysis were split into five datasets using VCFtools (Danecek et al., 2011) to enable identification of outlier loci differentiating high and low WSC populations within pools. These datasets consisted of five VCF files with individuals split into their respective pools but with the Parent populations removed, resulting in a total of 935 individuals retained for outlier detection analyses. All VCF files were converted into PLINK format (BED, BIM and FAM files) using PLINK v 1.9 (Purcell et al., 2007). The R package “*pcadapt*” v 4.0.3 was used to detect loci driving variation on the principal components (Luu et al., 2017). The *K_PC_* value (number of principal components) with the best fit to the data was determined using the scree test (Cattell, 1966) and interpretation of score plots with *K_PC_* values higher than those determined by the scree test method. To determine the number of principal components (*K_PC_*) separating high and low WSC populations, each of the five pools was analysed in PCAdapt separately. Scree plots were produced (**Supplementary Figure 2**) and visually assessed for optimal *K_PC_* value (Cattell, 1966), with components retained from the steep portion of the curve, prior to inflection into a flat line. Outlier SNPs based on Mahalanobis distance (Luu et al., 2017) at the optimal *K_PC_* value were identified, after correcting for false positives using the Bonferroni correction in each pool. SNPs were visualised on Manhattan plots using “*qqman*” v 0.1.4 (Turner, 2018), with outliers shown as exceeding Bonferroni false discovery thresholds, of α = 0.01 and α = 0.05.

#### 2.6.2 BayeScan

The five VCF files were converted into BayeScan format in R using “*vcfR*” v 1.8.0, “*adegenet*” v 2.1.1, and “*hierfstat*” v 0.04-22 (Jombart, 2008; Goudet and Jombart, 2015; Knaus and Grünwald, 2017). Population structure analysis identified little genetic differentiation between generations within the low WSC and high WSC divergent selections, respectively. Therefore, for this analysis within each pool the two high WSC populations were merged and two low WSC populations were merged, resulting in five population pairs to be tested. The analysis was conducted separately for each pool so that SNPs under divergent selection could be traced back to each pool. BayeScan was run with default parameters (20 pilot runs with 5,000 iterations, followed by a burn-in of 50,000 iterations, and prior odds for the neutral model was 10). The *q*-value for each locus was calculated and a false discovery rate of α = 0.05 was used to determine significant outlier loci that had positive alpha values.

#### 2.6.3 KGD-F_ST_

The filtered VCF file was converted to a Reference Alternative file using the KGD *vcf2ra_ro_ao.py* python script, and a separate file containing individual and population information was constructed. The “*Fst.GBS.pairwise()*” function was used to calculate approximate mean F_ST_ for each SNP between each population pair, accounting for GBS read depth (Dodds et al., 2015). The five population pairs were tested and SNPs with F_ST_ values greater than 0.3 and present in more than two pools at that threshold were called as outlier SNPs. Manhattan plots were created as described above. Outlier SNPs from all three analyses (PCAdapt, BayeScan and KGD-F_ST_) were visualised in a Venn diagram using the R package “*VennDiagram*” v 1.6.20 (Chen and Boutros, 2011). The scripts used to perform the outlier detection analyses can be found at https://github.com/SofiePearson/White_Clover_WSC_Outlier_Detection_GWAS.

### 2.7 Genome-wide association

A mixed-linear model implemented in the R package “*rrBLUP*” (Endelman, 2011) was used for an association analysis on a subset of individuals (*n* = 605) possessing both genotypic and phenotypic information (**Supplementary Figure 3**). Markers for this analysis were filtered to retain those with ≤ 50% missing data before the “*A.mat()*” function was used to impute missing values using the EM algorithm designed for GBS markers (Poland et al., 2012a). This resulted in 5,757 SNPs used in the analysis. Population structure and family relatedness was accounted for with “n.PC = 2” and a kinship matrix calculated by rrBLUP from the genotypic data. To account for multiple testing, a Bonferroni correction was applied and markers passing the threshold at an α of 0.05 were considered statistically significant (Bonferroni, 1936). Manhattan and Quantile-Quantile (Q-Q) plots were created for each phenotypic trait: leaf area, soluble sugars and starch (SSS), water-soluble carbohydrate (WSC) and other nutritional attributes including ash, crude protein, neutral detergent fibre, acid detergent fibre and lipid content. The R script used to perform GWAS can be found at https://github.com/SofiePearson/White_Clover_WSC_Outlier_Detection_GWAS.

### 2.8 Changes in genotypes due to selection over time

As a complement to the methods described above, changes in genotype frequencies from generation to generation were evaluated. Each outlier SNP detected by ≥2 outlier analyses, in each population, was assessed in this way. Genotype proportions for each SNP were extracted using VCFtools v 0.1.16 –*extract-FORMAT-info* GT and patterns were investigated (Danecek et al., 2011).

### 2.9 Identification of candidate genes

The physical positions of outlier SNPs identified by ≥2 outlier analyses were used to locate potential candidate genes. For outlier SNPs located to introns or exons, the host gene was recorded as the best candidate. SNPs identified in coding regions of a gene were investigated further to determine if they were likely to affect protein function or structure. Geneious Prime v 2019.1.1 (http://www.geneious.com/) was used to determine the position of the SNP in the protein and whether there was a synonymous or non-synonymous change. For outlier SNPs that occurred outside of genes, a maximum distance of 10 Kbp either side of each SNP were recorded. When considering potential candidate genes, the upstream and downstream regulatory elements of the gene, including the promoter region, were also considered. If intergenic outlier SNPs were located less than 1 Kbp away from the start codon of a gene, they were also classified as putatively in linkage disequilibrium with the gene due to the proximity to a promoter. White clover genome annotations (Griffiths et al., 2019), BLAST (Johnson et al., 2008), UniProt (The Uniprot Consortium, 2018) and STRING v 11.0 (Szklarczyk et al., 2019) were used to identify genes and their functions.

## 3 Results

### 3.1 Phenotypic variation

#### 3.1.1 Water-soluble carbohydrate phenotyping

**Figure 2:**
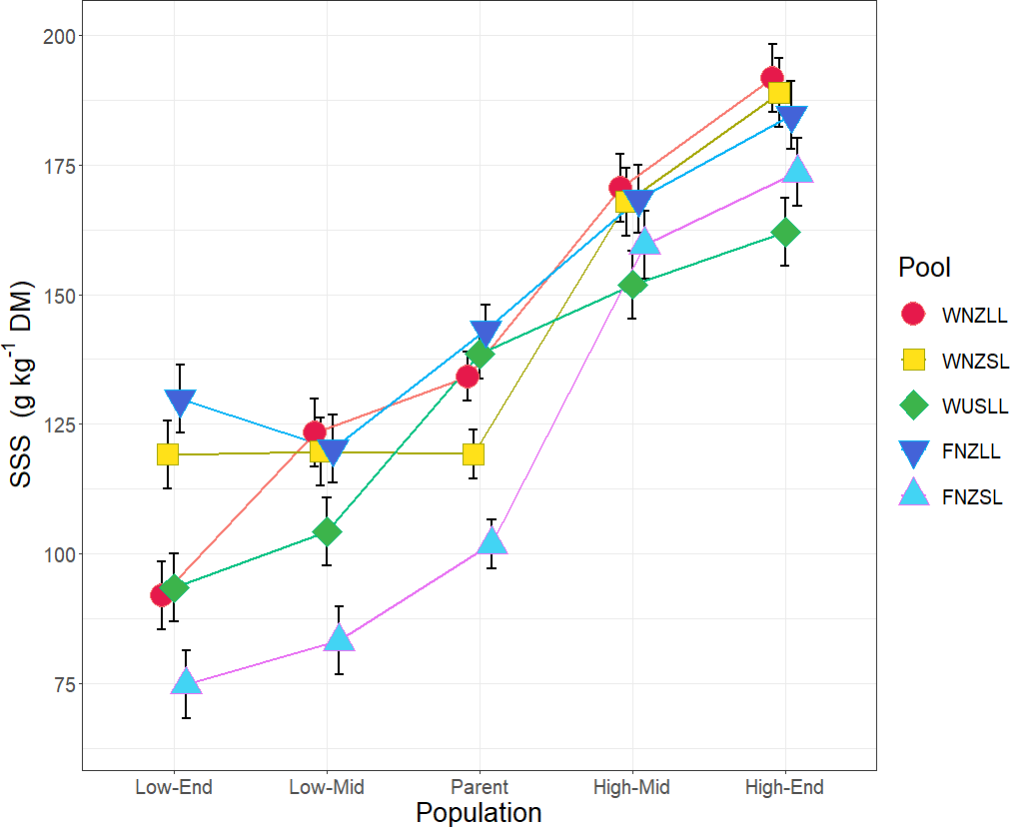
Population fitted values (adjustment for treatment, block, row and column effects) and standard error, for soluble sugars and starch (SSS) as a measure of water-soluble carbohydrate (WSC). Populations are grouped by pool as indicated by colour and symbol combinations. The *x*-axis indicates a timeline by generation, where: Low, low WSC; High, high WSC; End, End generation; Mid, Middle generation; Parent, Parent generation. Population means for SSS are based on *n* = 20 – 40.

Water-soluble carbohydrate (WSC) content was measured using two NIRS calibrations (soluble sugars and starch, SSS; and WSC-NIRS), which were found to be highly correlated within the sample set (r^2^ = 0.92, *p* < 0.0001), and therefore subsequent analyses focused principally on SSS alone. Population fitted values for SSS are presented in **Figure 2**. Comparison of population fitted values within each pool showed an overall trend for SSS to increase by selection cycle for high WSC selections and, conversely, decrease by selection cycle for low WSC selections (**Table 1**). When averaged across all pools, there were significant differences (*p* < 0.01) for all comparisons, with the low WSC populations lower for SSS than the Parent, and the high WSC populations higher for SSS content than the Parent (**Table 1**). Within individual pools, high and low WSC populations did not always differ significantly from the Parent population, but in all pools there were significant (*p* < 0.05) differences between the Low-End and High-End populations (mean difference of 78.3 g kg^-1^ DM) (**Table 1**). These results confirm that breeding for divergent WSC in the five pools was successful, with a mean 76.9% difference in SSS between the Low-End and High-End populations (**Supplementary Table 1**).

**Table 1:** Estimated phenotype means for each divergently-selected population compared to the parental mean after adjusting for treatment, block, row and column effects.

	SSS (g kg^-1^ DM)			Leaf area (cm^2^)		
Comparison	Difference	SE	*p*-value	Difference	SE	*p*-value
WNZLL-Low-End – WNZLL-Parent	-42.2	7.77	<0.01**	-0.83	0.16	1
WNZLL-Low-Mid – WNZLL-Parent	-10.9	7.78	0.97	0.6	0.16	1
WNZLL-High-Mid – WNZLL-Parent	36.4	7.77	<0.01**	2.74	0.16	0.37
WNZLL-High-End – WNZLL-Parent	57.5	7.77	<0.01**	2.92	0.16	0.27
WNZSL-Low-End – WNZSL-Parent	-0.098	7.79	1	0.56	0.16	1
WNZSL-Low-Mid – WNZSL-Parent	0.44	7.78	1	1.81	0.16	0.85
WNZSL-High-Mid – WNZSL-Parent	48.7	7.76	<0.01**	5.89	0.16	<0.01**
WNZSL-High-End – WNZSL-Parent	69.6	7.78	<0.01**	5.48	0.16	<0.01**
WUSLL-Low-End – WUSLL-Parent	-45.1	7.8	<0.01**	-4.41	0.17	0.02*
WUSLL-Low-Mid – WUSLL-Parent	-34.2	7.77	<0.01**	-1.65	0.16	0.99
WUSLL-High-Mid – WUSLL-Parent	13.4	7.76	0.82	1.14	0.16	1
WUSLL-High-End – WUSLL-Parent	23.6	7.76	0.054	-0.87	0.16	1
FNZLL-Low-End – FNZLL-Parent	-13.4	7.77	0.82	-5.37	0.16	<0.01**
FNZLL-Low-Mid – FNZLL-Parent	-22.8	7.79	0.07	-3.49	0.16	0.15
FNZLL-High-Mid – FNZLL-Parent	25.2	7.78	0.029*	0.72	0.16	1
FNZLL-High-End – FNZLL-Parent	41.4	7.79	<0.01**	1.19	0.16	1
FNZSL-Low-End – FNZSL-Parent	-27.1	7.76	0.012*	-1.5	0.16	0.94
FNZSL-Low-Mid – FNZSL-Parent	-18.7	7.77	0.29	0	0.16	1
FNZSL-High-Mid – FNZSL-Parent	57.8	7.78	<0.01**	-0.75	0.16	1
FNZSL-High-End – FNZSL-Parent	71.6	7.77	<0.01**	-1.77	0.16	0.8
Low-End – Parent	-25.6	3.47	<0.01**	-2.18	0.07	<0.01**
Low-Mid – Parent	-17.2	3.49	<0.01**	-0.43	0.07	1
High-Mid – Parent	36.7	3.46	<0.01**	1.95	0.07	0.01**
High-End – Parent	52.7	3.48	<0.01**	1.35	0.07	0.24
WNZLL-High-End – WNZLL-Low-End	99.6	8.99	<0.01**			
WNZSL-High-End – WNZSL-Low-End	69.7	8.98	<0.01**			
WUSLL-High-End – WUSLL-Low-End	68.7	8.98	<0.01**			
FNZLL-High-End – FNZLL-Low-End	54.8	8.98	<0.01**			
FNZSL-High-End – FNZSL-Low-End	98.7	8.98	<0.01**			

Low, low water-soluble carbohydrate (WSC), High, high WSC; Parent, Parent generation; Mid, Middle generation; End, End generation; W, Widdup; F, Ford; NZ, New Zealand/Aotearoa; US, United States of America; LL, large leaf; SL, small leaf and SE, standard error.

Significance codes: ** ≤ 0.01, * = 0.01 – 0.05, no symbol ≥ 0.05 at α = 0.05.

Soluble sugars and starch (SSS; grams per kilogram dry matter, g kg^-1^ DM) and leaf area (cm^2^) population fitted values compared to the Parent population are presented in the “Difference” column. For SSS, the High-End population fitted values are compared to the Low-End population fitted values for each pool and are also presented in the “Difference” column. Standard error and *p*-values are shown for each comparison. *p*-values are adjusted for multiple comparisons calculated using the R package “*emmeans*”.

#### 3.1.2 Leaf area phenotyping

Population fitted values for mean leaf area (cm^2^) are presented in **Supplementary Table 2**. When compared against Parent population values, there was a trend for leaf area to increase or decrease with WSC selection, but there were only four instances where that change was statistically significant (*p* < 0.05) (**Table 1**). The FNZLL (-5.4 cm^2^, *p* < 0.01) and WUSLL (-4.4 cm^2^, *p* = 0.02) pools showed a significant decrease in leaf area from the Parent to Low-End population. In the WNZSL pool there was a significant increase in leaf area in both the High-Mid and High-End populations relative to the Parent population (+5.9 cm^2^, *p* < 0.01 and +5.5 cm^2^, *p* < 0.01, respectively).

#### 3.1.3 Correlation and regression analysis between water-soluble carbohydrate and leaf area

Correlation analysis was used to measure the strength of relationship between SSS and leaf area for each pool and for the combined pool dataset (**Supplementary Figure 4**). Weak to moderate positive linear relationships between SSS and leaf area were observed in all pools (**Supplementary Figure 4**), significant at *p* < 0.05 except for FNZSL (*p* = 0.73). Pearson’s coefficients of determination for WNZLL (r^2^ = 0.13), WNZSL (0.33), WUSLL (0.26), FNZLL (0.09), FNZSL (-0.015) and the combined dataset (0.14), indicated between 1.5 – 33% of the observed SSS phenotypic variation was accounted for by leaf area in each of the pools. These low r^2^ values demonstrate that the basic linear model (SSS the dependent variable and leaf area the independent variable) provided a poor to average fit to the data.

The data were then split into populations for each pool and regression analysis was used to test if leaf area was significantly predictive of SSS for each pool at the population level. Linear models were constructed with SSS as the dependent variable and the interaction between leaf area and population was used as the independent variable. Including populations in the linear model increased the adjusted r^2^ values up to 94 – 97% (**Supplementary Table 3**). This indicated that splitting the data into populations for each pool and analysing separately provided a better fit to the data than combining all population data points within each pool. The majority of slope coefficients for each pool (**Supplementary Figure 5** and **Supplementary Table 3**) were gradual and not significant (*p* > 0.05), with the exception of WUSLL-Parent (slope = 0.37, *p* = 0.004), FNZLL-Low-End (slope = 0.44, *p* = 0.037) and FNZLL-High-End (slope = 0.21, *p* = 0.04). Because all populations, except WUSLL-Parent, had non-significant *p*-values (p > 0.05) for both the intercept and slope, we were unable to reject the null hypothesis, allowing the conclusion that there was no relationship between SSS and leaf area for all but one population (WUSLL-Parent).

### 3.2 Genotyping

#### 3.2.1 DNA isolation, genotyping-by-sequencing library evaluation and single nucleotide polymorphism filtering

High molecular weight (> 15 Kbp) genomic DNA and free from RNA contamination, was isolated successfully from 1,536 plants. For GBS library construction, 47 individuals per population with DNA concentration > 10 ng µL^-1^ were selected (*n* = 1,175 total). Bioanalyzer evaluation of the 13 pooled GBS libraries, prior to sequencing, showed that small adapter dimers present in the pre-size selection libraries (88 bp) were removed successfully post-size selection and that library fragment sizes were limited to the targeted 193 – 313 bp range.

A total of 191,484 SNPs were called initially across all samples. After filtering for depth, multiallelic loci, missing and minor allele frequency, a total of 14,743 SNPs were retained for 1,113 samples. A total of 109 samples were removed across all populations, most of which were population WNZSL-Parent, due to a high proportion of missing data (> 80%). A further 15 samples from amongst other populations were removed due to high missing data (> 80%). Positive control samples, a single genotype repeated in all 13 GBS libraries, were at first retained to check for consistency across GBS libraries using a principal component analysis (PCA). Duplicated GBS data from 47 individuals were also removed as they were derived from duplicated technical replicates included for quality control and were not required for subsequent analyses.

#### 3.2.2 Single nucleotide polymorphism distribution and density

The number of SNPs found on each pseudomolecule and the SNP density across the white clover reference genome was investigated using the 14,743 SNPs from 1,113 samples identified above. Pseudomolecules were assigned to their relevant subgenomes, where pseudomolecules 1 to 8 belong to the *T. occidentale*-derived (Tr_To_) subgenome, and pseudomolecules 9 to 16 belong to the *T. pallescens*-derived (Tr_Tp_) subgenome (Griffiths et al., 2019). The number of SNPs per pseudomolecule was strongly correlated to pseudomolecule length (r^2^ = 0.96, **Supplementary Figure 6**), with higher numbers of SNPs on the longer pseudomolecules, demonstrating that the SNPs are evenly distributed across the genome. A mean of 16.1 (± 1.6 standard deviation) SNPs per Mbp was found across all pseudomolecules. The lowest SNP density was found on pseudomolecules 14, 10 and 6 with 12.5, 13.9 and 14.5 SNPs per Mbp, respectively. The highest densities were on pseudomolecules 5, 3 and 4 with 19.1, 18.0 and 17.7 SNPs per Mbp, respectively.

### 3.3 Population structure

#### 3.3.1 Discriminant analysis of principal components

**Figure 3:**
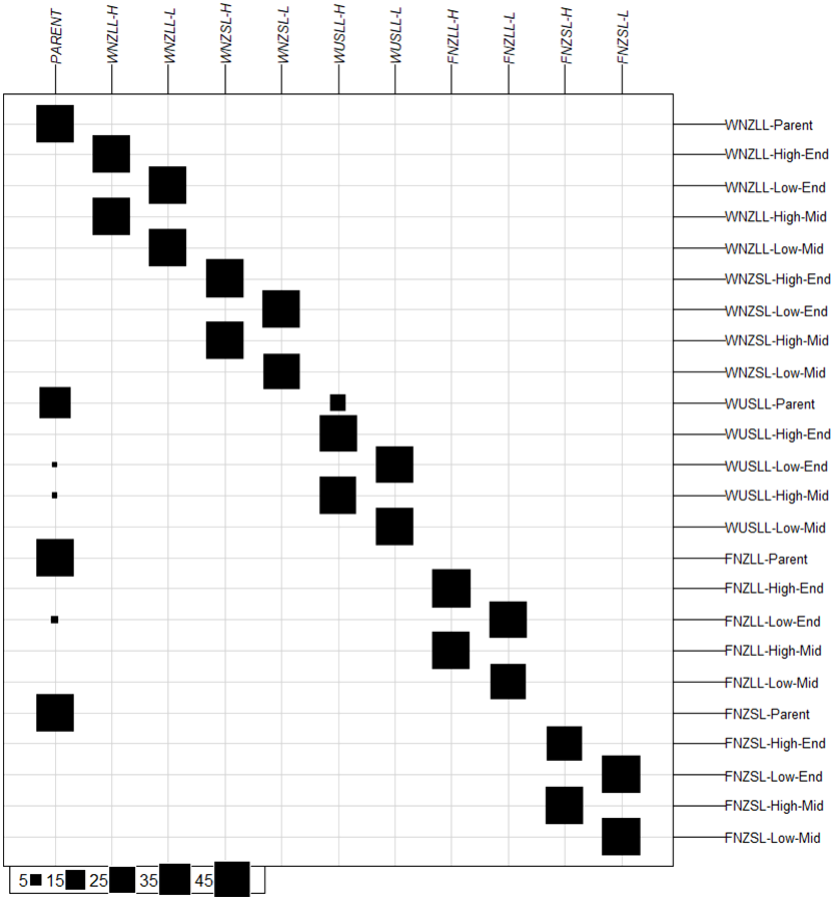
Group assignment based on *K*-means clustering prior to discriminant analysis of principal components for 24 populations. Original populations are positioned horizontally, and *K*-means determined clusters are positioned vertically. Size of black boxes represent the number of individuals assigned to the *K*-means determined cluster from the original population, with the scale presented in the bottom left-hand corner. High, high water-soluble carbohydrate (WSC); Low, low WSC; Parent, Parent generation; E, End generation; and M, Middle generation.

Discriminant analysis of principal components (DAPC) was conducted to determine the number of clusters described by the data and to validate the pre-defined genetic clusters (i.e., populations within pools). The lowest Bayesian information criterion (BIC) value from the “*find.clusters()*” function corresponded to *K* = 11 (**Supplementary Figure 7**) which was therefore selected as the number of clusters described by the data. The assignment of individuals to the 11 clusters was compared with the *a priori* population grouping (**Figure 3**). In the following, the names of the 11 DAPC clusters are italicised and the 24 a *priori* population names are non-italicised. Individuals in both high WSC populations within a pool tended to group together in a single cluster, e.g., WNZLL-High-Mid and WNZLL-High-End comprised the *WNZLL-H* cluster, as did individuals from low WSC *a priori* populations e.g., WNZLL-Low-Mid and WNZLL-Low-End comprised the *WNZLL-L* cluster. Individuals from all the Parent populations grouped in a single cluster (*PARENT*), except for nine individuals from the WUSLL-Parent population that grouped with *WUSLL-H*. One to two samples from the FNZLL-Low-End, WUSLL-High-Mid and WUSLL-Low-End also grouped with the *PARENT* cluster.

**Figure 4:**
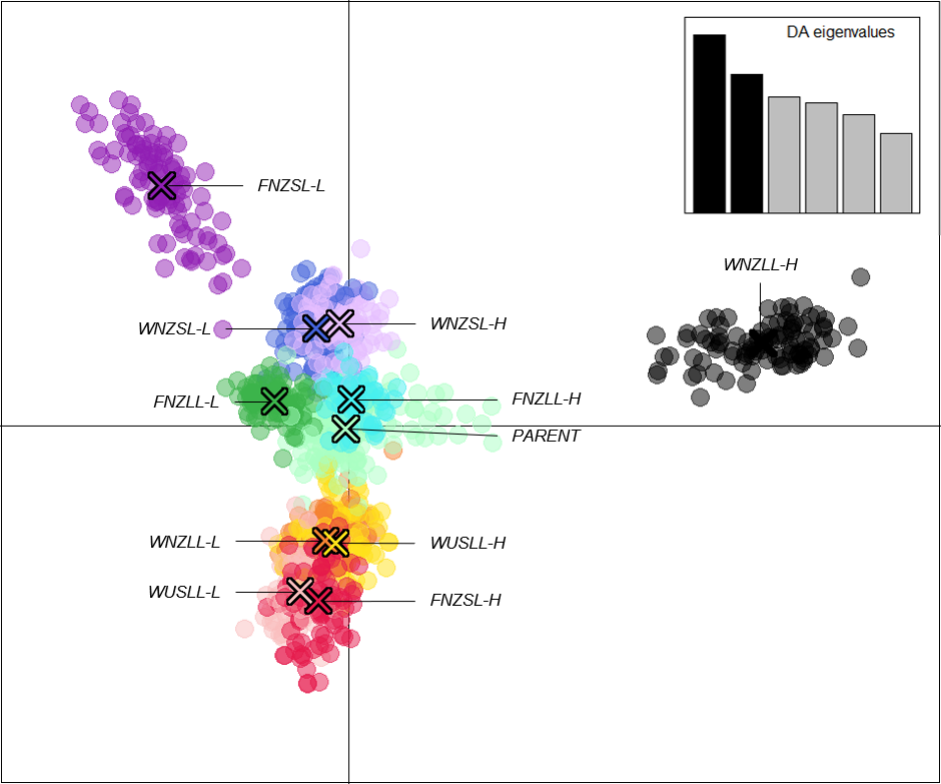
Discriminant analysis of principal components (DAPC) scatter plot of 1,113 individuals using 14,743 SNPs based on 11 assigned genetic clusters. Six principal components (**Supplementary Figure 8**) and six discriminant functions (DFs) were retained for analyses to describe the relationship between the genetic clusters. The scatter plot shows the first two DF from the DAPC analysis (X and Y axis explaining 24.6% and 19.3% of genetic variance, respectively) with the scree plot of eigenvalues of the linear discriminant analysis (LDA) shown in the inset. Populations are labelled and colour coded at *K* = 11 as determined from the *K*-means clustering algorithm. Each dot represents a single individual and the centre of each cluster, as determined by a minimum spanning tree based on the squared distances between populations, is indicated by a cross.

The “*xvalDapc()*” function determined the optimal number of PCs to retain was 43 (root mean square error = 0.0033). However, both the root mean square error and mean successful assignment plateaued at 6 PCs (root mean square error = 0.0107 and mean successful assignment = 0.9928) with very little change thereafter (**Supplementary Figure 8**), therefore DAPC was run with 6 PCs retained. A scatter plot showing the 11 clusters inferred by *K*-means and the two axes representing the first two discriminant functions (DFs) of the DAPC analysis (**Figure 4**). The first DF showed a general separation of high WSC and low WSC populations with *High* clusters centred to the right of the plot, *Low* clusters centred to the left, and the *PARENT* plants clustering in the middle of the plot. The *WNZLL-H* and *FNZSL-L* clusters were clearly isolated from the bulk of the clusters. With respect to their counterpart populations (*WNZLL-L* and *FNZSL-H*), separation occurred on both the first and second DF. For *WNZSL* and *WUSLL*, Low and High population clusters showed very little separation on the first two DF and in fact clear separation was not observed on any DF (data not presented). The *FNZLL-L* and *FNZLL-H* clusters only showed clear separation on the fifth DF.

#### 3.3.2 Pairwise F_ST_

Pairwise fixation index (F_ST_) was calculated both for the *a priori* population grouping (*K* = 24) and for the grouping identified above (*K* = 11). For biallelic marker systems Wright (1978) suggests that F_ST_ values from 0 – 0.05 indicate little differentiation, 0.05 – 0.15 moderate differentiation, 0.15 – 0.25 great differentiation, and values above 0.25 indicate high differentiation (Balloux and Lugon-Moulin, 2002; Hartl and Clark, 2007). For the *a priori* population grouping (*K* = 24), the WUSLL-Parent population showed moderate genetic differentiation from the NZ Parent populations (0.06 – 0.08), while F_ST_ values were low among the three NZ Parent populations (0.03 – 0.04). Pairwise comparisons of the high WSC populations (Mid and End) within each pool and of the low WSC populations (Mid and End) within each pool showed low to moderate differentiation, with F_ST_ values ranging from 0.03 – 0.09. Divergent low and high WSC pairs within a pool (e.g., WNZLL-Low-Mid compared with WNZLL-High-Mid) showed greater differentiation as indicated by an F_ST_ range of 0.12 – 0.22 (**Supplementary Table 4**). Pairwise F_ST_ values based on the 11 *K*-means determined clusters showed moderate genetic differentiation between *High* and *Low* clusters within each pool, ranging between 0.13 – 0.19. Very low genetic differentiation was observed between the *PARENT* cluster and all the other clusters (0.07 – 0.10) (**Supplementary Table 5**).

#### 3.3.3 Analysis of molecular variance

A total of 342 loci with missing values less than 5% were used for the analysis of molecular variance (AMOVA). The AMOVA for *K* = 24 revealed that most genetic variation was partitioned within populations (80.7%), and the remainder partitioned among populations (19.3%). A hierarchical AMOVA for the 11 genetic clusters determined by *K*-means revealed that 15.4% of the variance was distributed among clusters, and only 5.4% was distributed among populations within clusters. Approximately similar variance was found within populations or clusters (*K* = 24: 80.7%, p < 0.001; *K* = 11: 79.2%, p < 0.001), indicating that genetic variation is mainly distributed within populations (**Supplementary Table 6**). Each of the DAPC, pairwise F_ST_ and AMOVA results indicate that, genetically, the two high WSC populations (High-Mid and High-End) for each pool can be grouped together and the two low WSC populations (Low-Mid and Low-End) for each pool can be grouped together. Population groupings for subsequent outlier locus detection were based on this *K* = 10 grouping (Parent populations were excluded).

### 3.4 Outlier loci detection

**Figure 5:**
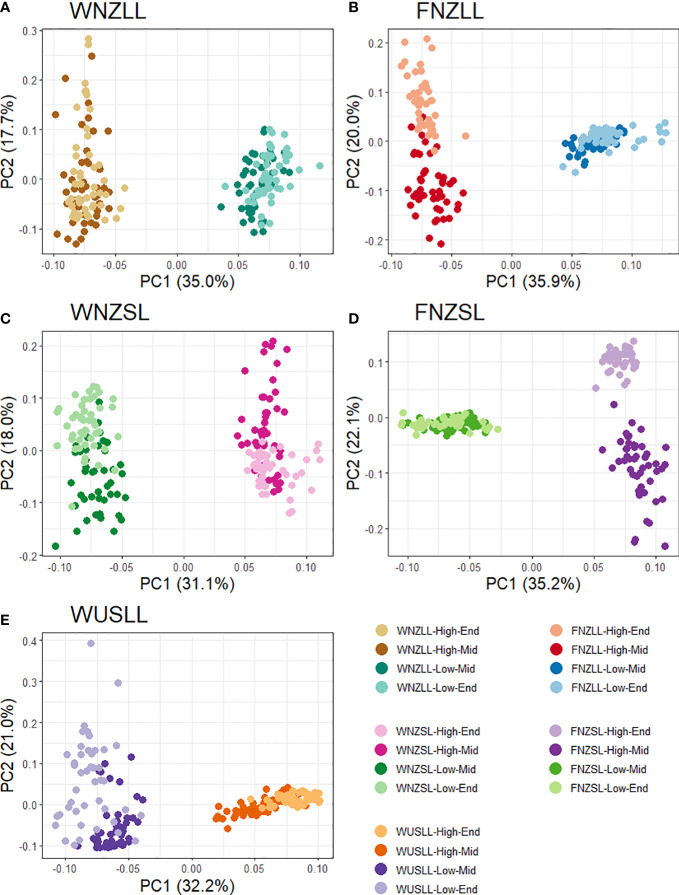
Score plots from PCAdapt analysis using the first two principal components (PC) for all five pools. Each dot represents an individual and the colour corresponds to individuals from the same population. Each pool has four populations as the Parent populations were excluded from the analysis. A total of 188 individuals were used from the WNZLL pool **(A)**, 186 from WNZSL **(B)**, 195 from WUSLL **(C)**, 182 from FNZLL **(D)** and 184 from FNZSL **(E)**, for a combined total of 935. Population information is displayed in the key in the bottom right corner.

PCAdapt was used to identify SNPs corresponding to PCs that differentiate low and high WSC populations, within each pool. Cattell’s scree test (**Supplementary Figure 2**) and interpretation of score plots (**Supplementary Figure 9**), determined that the *K_PC_* value, the number of PCs to investigate, in all four pools was *K_PC_* = 1. The first PC captures the distinction between high and low WSC populations in all pools (**Figure 5**). Therefore, to identify SNPs related to WSC, we focused on the SNPs associated with PC1 only. The number of SNPs used for outlier detection in PCAdapt ranged from 10,976 to 11,479 per pool, with a mean of 11,133. The genome-wide significance thresholds were determined for each pool at Bonferroni false discovery thresholds of α = 0.01 and α = 0.05, using their respective total number of SNPs (**Supplementary Table 7**). The average *p*-value threshold used for outlier detection at α = 0.05 was 4.49e-06, which is 5.34 on the log scale. Any SNPs associated with PC1 with -log_10_(*p*-values) larger than 5.34 were retained from each pool and identified as putative outliers. To reduce the risk of detecting SNPs associated with population structure, SNPs identified as outliers were subjected to another criterion: they had to be present as outliers in two or more pools. Of 643 total outlier SNPs detected using PCAdapt, 36 were found in common amongst two or more pools based on PC1. A total of 329 outliers were detected using BayeScan at α = 0.05, with 27 common to two or more pools (**Supplementary Table 8**). All outliers identified by BayeScan exhibited positive alpha values, indicating that only SNPs putatively under directional selection were detected. The KGD-F_ST_ method detected the largest number of outliers of the three methods, with 1,188 in total and 229 in common amongst two or more pools (**Supplementary Table 8**). The strongest candidates for selection were 33 SNPs found using two or more of the outlier detection methods (**Supplementary Figure 10**). The two F_ST_ based methods (BayeScan and KGD-F_ST_) had the most SNPs in common (*n* = 22) whereas PCAdapt and BayeScan only had two SNPs in common and PCAdapt and KGD-F_ST_ had 13 SNPs in common.

Of the 33 candidate SNPs, five were found in exons, 15 were in introns and the remainder were either intergenic or in promoter regions. One of the exon-located SNPs exhibited a synonymous mutation, while the other four had non-synonymous mutations leading to a change in amino acid (**Supplementary Table 9**). Seven of the SNP-associated genes had unknown functions, while the remaining 23 had putative functions (**Supplementary Table 9**). One SNP, 16_32428574, was identified by BayeScan and KGD-F_ST_ in the WNZLL and FNZSL pools and found in the intron of *ERD6-like 4*, a gene coding for a sugar transporter located on the vacuole membrane.

To assess the potential that SNP genotypes changed due to random genetic drift, generational changes in genotype and allele frequencies of the 33 candidates were investigated. The vast majority of the 33 SNPs identified as outliers demonstrated a complete sweep where fixation of the reference allele occurred in the high WSC populations and the alternate allele became fixed in the low WSC populations, within the first two to three generations (**Supplementary Table 10**). This was observed for all 33 SNPs in two or more pools. There were instances where fixation was not achieved but genotype frequencies showed an apparent directional shift across generations. For example, in both the WUSLL and FNZLL pools at SNP 2_6673787 (**Supplementary Table 10**) allele frequencies were fixed for the reference allele in the low WSC populations but there was a transition from Low-End and Low-Mid to the Parent populations and then to the High-Mid and the High-End populations whereby the alternate allele increased in frequency over successive generations.

### 3.5 Genome-wide association study

**Figure 6:**
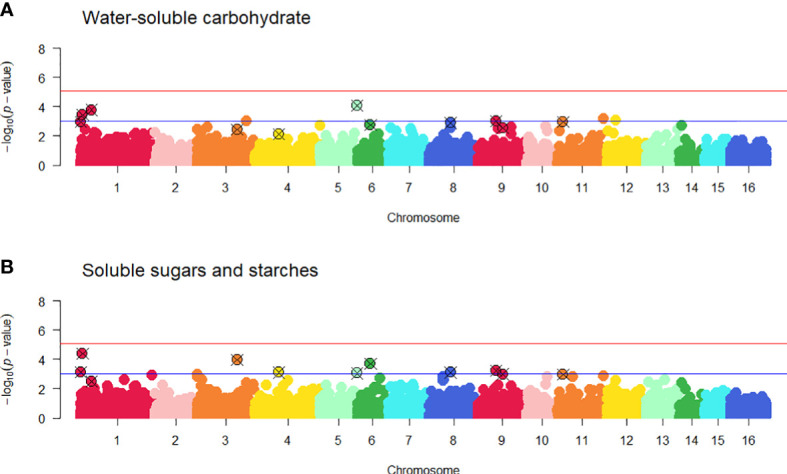
Manhattan plots from the genome-wide association study (GWAS) of water-soluble carbohydrate, WSC-NIRS **(A)** and soluble sugars and starch, SSS **(B)** using 5,757 SNP markers and 605 individuals. -log_10_(*p*-values) are plotted against physical map position of SNPs with subgenomes of corresponding chromosomes (i.e., pseudomolecules) similarly coloured (Tr_To_ 1 – 8 and Tr_Tp_ 9 – 16). Significant loci lie above the false discovery rate thresholds as denoted by the red (α = 0.01) and blue (-log_10_(*p*-value) > 3) solid lines. Twelve SNPs with -log_10_(*p*-values) > 3 identified for the WSC-NIRS and SSS traits are highlighted. Quantile-Quantile plots for each trait are presented in **Supplementary Figure 11**.

A genome-wide association study (GWAS) was carried out using 24 white clover populations that had both genotype and WSC phenotype data. No SNPs were found to be significantly associated with SSS after correction for multiple testing (**Figure 6** and **Supplementary Figure 11**). However, ten SNPs on pseudomolecules 1, 3, 4, 6, 8, 9 and 11 were ranked highly for the SSS trait, and close to the false discovery threshold with -log_10_(*p*-values) > 3. By the same criterion two additional SNPs on pseudomolecules 1 and 5 were ranked highly for the WSC-NIRS trait but not SSS (**Table 2**). On the white clover reference genome, one of the SNP markers on pseudomolecule 1 located to the coding region of a *VPS35B* (Vacuolar protein sorting-associated protein 35B) gene, the second located to the intron of a *CBP* (chlorophyll a-b binding protein) gene, and the third located to the coding region of a La-related protein 7-like gene. The two markers on pseudomolecule 3 located to the intron of a transcription regulating protein (PKS-NRPS hybrid synthetase CHGG_01239-like), and the marker on pseudomolecule 4 was located 305bp upstream of the start codon of a gene with unknown function. The SNP on pseudomolecule 5 was located 855 bp before a *glgC* (glucose-1-phosphate adenylyltransferase) gene, the SNP on pseudomolecule 6 was intergenic, the SNP on pseudomolecule 8 located to the intron of *COG8* (conserved oligomeric Golgi complex subunit 8), the SNPs on pseudomolecule 9 were located in the coding region of *UPL6* (E3 ubiquitin-protein ligase) gene and on the intron of a mixed-amyrin synthase gene. The SNP on pseudomolecule 11 located to the coding region of a clustered mitochondria protein gene. Two SNPs located in coding regions conferred non-synonymous mutations, as both altered the first base of a codon and subsequently the encoded amino acid. The SNP located in *VPS35B* caused an isoleucine to valine change, while the SNP in *UPL6* caused a glutamine to glutamic acid change. The SNP in the coding region for the clustered mitochondrial protein gene and the SNP for the La-related protein 7-like gene both conferred synonymous mutations. The SNP on pseudomolecule 5 was located near a gene (*glgC*) that may be considered a prime candidate for a role in WSC accumulation, based on its inferred function. Significant SNPs for other phenotypic traits are found in **Supplementary Table 11** and visualised in **Supplementary Figure 12**.

**Table 2:** Genome position and gene annotation for SNPs with large -log_10_(*p*-values) identified from a genome-wide association study (GWAS).

Pseudomolecule	SNP position (bp)	-log_10_(*p*-value) for WSC-NIRS	-log_10_(*p*-value) for SSS	Genomic region	Gene model identifier and Gene annotation (Ann)	Potential function of gene and codon change
1 (Tr_To_ – 1)	102,715	2.93	3.14	Intron	chr1.jg10.t1Ann: Chlorophyll a-b binding protein (*Trifolium pratense*)	Photosynthesis, light harvesting in photosystem I.
1 (Tr_To_ – 1)	2,338,028	3.51	4.52	Exon	chr1.jg302.t1Ann: VPS35B Vacuolar protein sorting-associated protein 35B (*Arabidopsis thaliana*)	Protein storage and vacuole biogenesis. Retarding the senescence of leaves. ATC to GTC changes isoleucine to valine.
1 (Tr_To_ – 1)	13,746,102	3.99	2.70	Exon	chr1.jg1888.t1Ann: La-related protein 7-like, partial (*Trifolium pratense*)	RNA processing GTG to GTC no change
3 (Tr_To_ – 3)	51,874,121 51,874,123	2.39	3.97	Intron	chr3.jg7956.t1Ann: PKS-NRPS hybrid synthetase CHGG_01239-like (*Cicer arietinum*)	Positive regulation of transcription from RNA polymerase II promoter in response to iron ion starvation.
4 (Tr_To_ – 4)	30,841,647	2.38	3.44	Promoter	305 bp from start codon of chr4.jg4214.t1Ann: Protein with unknown function/hypothetical protein MTR_6g072130 *(Medicago truncatula*)	
5 (Tr_To_ – 5)	47,903,593	3.83	2.85	Promoter	855 bp upstream from start codon of chr5.jg7106.t1Ann: *glgC* Glucose-1-phosphate adenylyltransferase small subunit 1, chloroplastic (*Vicia faba*)	Starch biosynthesis and glycan biosynthesis.
6 (Tr_To_ – 6)	16,865,077	3.01	3.94	Intergenic		
8 (Tr_To_ – 8)	27,636,527	2.79	3.01	Intron	chr8.jg3785.t1Ann: *COG8* conserved oligomeric Golgi complex subunit 8 (*Medicago truncatula*)	Intra-Golgi vesicle-mediated transport and protein transport.
9 (Tr_Tp_ – 1)	23,070,656	2.99	3.19	Exon	chr9.jg3440.t1Ann: UPL6 E3 ubiquitin-protein ligase UPL6 (*A. thaliana*)	Protein post-translational modifications.Response to water deficit and cold stress. CAA to GAA changes glutamine to glutamic acid.
9 (Tr_Tp_ – 1)	31,736,793	2.65	3.18	Intron	chr9.jg4697.t1Ann: Mixed-amyrin synthase (*Pisum sativum)*	Pentacyclic triterpenoid biosynthetic process, alpha- and beta-amyrin synthase activity.
11 (Tr_Tp_ – 3)	7,689,073	3.29	3.36	Exon	chr11.jg1201.t1Ann: Clustered mitochondria protein (*Cicer arietinum*)	Translational initiation ACT to ACC no change

A total of 605 individuals were used for the GWAS with a mean of 25 individuals per population (*n* = 24). A total of 122 individuals were used from the WNZLL pool, 83 from WNZSL, 136 from WUSLL, 127 from FNZLL and 137 from FNZSL.

bp, base pairs; WSC-NIRS, water-soluble carbohydrate by NIRS; SSS, Soluble sugars and starch by NIRS; Tr_To_, white clover Trifolium occidentale-derived subgenome; Tr_Tp_, white clover T. pallescens-derived subgenome.

## 4 Discussion

This study substantially increases our knowledge of genomic regions and candidate genes underpinning WSC accumulation in white clover leaves, by utilising GBS and phenotypic data from a population resource in which divergent recurrent selection for foliar WSC was undertaken over multiple generations.

### 4..1 The association between selection for WSC and change in leaf size

Phenotypic analysis of foliar WSC in five breeding pools confirmed breeding progress, showing that populations with divergent WSC phenotypes had been generated in each of the breeding pools. For the purpose of characterising the genetic control of foliar WSC *per se*, it is crucial to account for any effects of leaf size. Woodfield et al. (2001) reported that large-leaved white clover types tended to have higher levels of WSC than medium- and small-leaved cultivars, highlighting a potential positive correlation between the two traits and suggesting that gains in foliar WSC could be achieved by breeding for increased leaf size. However, an inverse relationship exists between leaf size and stolon density, a trait that influences vegetative persistence (Charlton and Stewart, 1999) meaning that breeding for larger leaves to increase WSC content could negatively impact sward persistence (Jahufer et al., 1999; Woodfield and Clark, 2009). In the current study, only weak correlations between WSC and leaf size were observed, in some pools, and once population was accounted for in the linear models there was little relationship between the two variables. The association between leaf area and WSC has not been researched extensively in legumes, and the outcomes are not consistent. For example, in lentil, a weak negative relationship between WSC and leaf area was identified (Tahir et al., 2019), whereas either positive (Malinowski et al., 1998; Woodfield et al., 2001) or no association (Ruckle et al., 2018) has been identified in white clover. Similarly, Ruckle et al. (2017) reported little to no correlation between biomass and starch concentration in red clover. The current study suggests that white clover plants with small leaves can accumulate WSC to levels comparable with large-leaved plants, as demonstrated in **Figure 2**, and that increased WSC content can be achieved without changing leaf size classes. We conclude that, although there was variation in leaf size in some of the breeding pools, changes in leaf WSC did not result from indirect selection for leaf size.

### 4.2 Population genetic structure

Assessment of population structure, which reflects relatedness among samples, is required in outlier detection and GWAS analyses as it can be a confounding factor - population structure can result in strong signals being assigned to SNPs that are not associated with the trait of interest (Sul et al., 2018), producing false positive associations. To investigate population structure and partitioning of genetic variation for the outlier detection methodologies, three analyses were used.

*K*-means clustering determined that the SNP dataset from the 24 populations described 11 genetic clusters in which the two high WSC populations (High-Mid and High-End) within each pool coalesced to a single cluster, as did the two low WSC populations (Low-Mid and Low-End). By contrast, the Parent populations for all pools formed a single cluster. AMOVA on this reduced genetic cluster set showed greater variation among clusters (15.4%) than among populations within clusters (5.4%), indicating the populations within clusters were very similar. This is supported by F_ST_ values (F_ST_ 0.03 – 0.09) representing low genetic differentiation (Wright, 1978) between populations within clusters and higher values (F_ST_ 0.12 – 0.22) between Low WSC and High WSC clusters, indicating selection was successful in driving genetic separation between the high and low WSC populations. Selection within the first 2 – 3 generations (Parent to Mid), therefore, produced the greatest genetic change, with less change occurring in the next 2 – 3 generations (Mid to End). This pattern is reflected in both WSC phenotypes (**Figure 2**) and genotype frequencies of outlier SNPs. A mean difference of 36 g SSS kg^-1^ DM occurred between Parent and High-Mid populations, compared with 16 g SSS kg^-1^ DM between High-Mid and High-End populations and, similarly a -17.24 g SSS kg^-1^ DM differential occurred between Parent and Low-Mid populations compared with -8.34 g SSS kg^-1^ DM between Low-Mid and Low-End populations (**Table 1**). From a heterozygous state in the Parent population most outlier SNPs exhibited rapid fixation for one allele in the Mid populations, and no further changes were observed between the Mid and End populations (**Supplementary Table 10**). Thus, selection for high WSC white clover individuals can be achieved in a short time frame of 2 – 3 generations and for the purposes of identifying SNPs under selection for divergent foliar WSC, the population grouping that showed the most genetic differentiation (*K* = 11) should be used. One SNP associated with movement of sugars showed this pattern (**Supplementary Table 10**) and is discussed in greater detail below.

DAPC placed all Parent populations into a single cluster, suggesting a lack of population structure at the parental source material level. However, pairwise F_ST_ analysis showed material from the Widdup US large leaf (WUSLL) pool was slightly more genetically distinct from the New Zealand (NZ) cultivars (F_ST_ 0.06 – 0.08), than the NZ Parent populations were among themselves (F_ST_ 0.03 – 0.04) (**Supplementary Table 4**). It has been demonstrated that white clover has extremely large effective population sizes worldwide and exhibits negligible population structure on continental and global scales (George et al., 2006; Olsen et al., 2007; Kooyers and Olsen, 2012; Kooyers and Olsen, 2013), with pairwise F_ST_ values < 0.03 in previous studies (Wright et al., 2017; Inostroza et al., 2018). It is, therefore, not surprising that while F_ST_ values were slightly higher between different countries of origin, these values were still very low. Furthermore, cultivars used in the WUSLL-Parent population included a mixture of US (e.g., ‘Tillman’ and ‘SRVR’; (Widdup et al., 2015)) and NZ material (e.g., ‘Grasslands Huia’ and ‘Ranger’; (Williams, 1983; Caradus et al., 1995; Widdup et al., 2010)). As the pedigrees of the Parent populations had cultivars in common (**Supplementary Table 12**), it may be expected that the Parent populations should not show a great level of genetic differentiation. All three methods suggest minimal population structure was observed in the 24 white clover populations. However, divergent selection has created a structure that differentiates high and low WSC populations and within the divergent lines there was low genetic variation.

Analysis of molecular variance (AMOVA) revealed that the genetic variation within each of the 24 white clover populations accounted for a mean 81% of the total variation, whereas 19% of the variation occurred among populations (**Supplementary Table 6**). These results align with previous AMOVA studies where 19 – 24% of genetic variation partitioned among white clover populations (Gustine and Huff, 1999; Khanlou et al., 2011; Collins et al., 2012). The high levels of intra-population diversity, as observed in the current study, may be attributed not only to white clover’s obligate outcrossing (Annicchiarico and Piano, 1995), which leads to genetically diverse populations with less genetic differentiation among populations (Hamrick and Godt, 1996; Nybom, 2004), but also other factors including a very recent human-associated range expansion (Zeven, 1991).

### 4.3 Outlier detection methodologies

After assessing population structure, three genome scan methods identified SNPs associated significantly with WSC levels that may also be linked to genes influencing this trait in white clover. The number of outlier SNPs differentiating high and low WSC populations varied among the methods, with few significant SNPs in common (**Supplementary Figure 10**). Unsurprisingly, the strongest overlap occurred between the two F_ST_-derived methods: BayeScan and KGD-F_ST_. However, BayeScan F_ST_ values were higher than those estimated by KGD-F_ST_, for all pairwise comparisons. For example, in the WNZLL pool, the minimum F_ST_ value determined for a SNP locus by BayeScan was 0.21 with a maximum of 0.69 and mean of 0.23. In the equivalent KGD-F_ST_ analysis the minimum F_ST_ value was 0.0, maximum of 0.99, and the mean was 0.04. Both sets of F_ST_ values fitted χ^2^ distributions (data not presented), but the mean BayeScan F_ST_ values were higher. BayeScan is relatively robust against confounding demographic processes, but strong selection, hierarchical structure, population bottlenecks and recent migration can impact this method and artificially inflate F_ST_ (Hermisson, 2009; Narum and Hess, 2011; Lotterhos and Whitlock, 2014; Lotterhos and Whitlock, 2015). Inflation of F_ST_ values due to population structure and a similar evolutionary history has been documented (Excoffier et al., 2009; Eckert et al., 2010b). Given the contrasting outcomes from BayeScan and KGD-F_ST_, it is likely that the inflated minimum F_ST_ values were due to analysing populations that are closely related. It is also not unusual for minimum F_ST_ values to sit around 0.2 when strong selection is occurring (Narum and Hess, 2011). For example, Reinert et al. (2019) presented F_ST_ values from a BayeScan analysis in barley that never dropped below 0.1. One of the benefits to BayeScan is that it runs two models for each locus: a neutral and a selection model. The posterior probability for both models is calculated, and the posterior odds are used as evidence for one model or the other. Log_10_(posterior odds) ≤ 2 indicates decisive evidence for selection and corresponds to large positive alpha values. Therefore, not only do the SNPs need to have high F_ST_ values, but also large alpha values to be indicative of adaptive selection. Although the inflated F_ST_ values technically indicate all the SNPs are under selection, as F_ST_ values greater than 0.25 indicate high genetic differentiation (Wright, 1978), the type of selection they are under can be determined from the alpha value. BayeScan calculates *q*-values for each locus, which is a test statistic directly related to the false discovery rate (FDR) and should be used to make decisions (Foll and Gaggiotti, 2008). Therefore, the F_ST_ values calculated by BayeScan in this study may be disregarded and focus should instead be placed on the alpha values of SNPs above the FDR threshold as indicated by the *q*-value, the approach implemented in the current study.

Most SNPs identified as outliers indicated a complete sweep, that is, fixation of one allele occurred in the high WSC populations and the alternate allele was fixed in the low WSC populations. This was often achieved within few generations as the Mid populations often showed fixation or near fixation for one allele. Because these SNPs exhibit clear changes in allele frequencies, multiple detection methods were able to detect these as outliers. BayeScan, however, appeared unable to detect outliers due to subtle changes in allele frequency such as occurs for an incomplete sweep. This was demonstrated in multiple pools, with SNPs 2_6673787, 4_9733285 and 12_3437942, as examples. These SNPs each showed gradual increases in reference allele homozygotes in the low WSC populations and alternate allele homozygotes in the high WSC populations, moving directionally from Mid to End populations. KGD-F_ST_ and PCAdapt detected these SNPs as outliers but BayeScan did not. In support of this observation, Narum & Hess (2011) showed that, under simulated strong selection, BayeScan could correctly identify all markers under selection. However, under weak selection, only two of five markers were correctly identified. In the current study, we aimed to maximise detection of SNPs under strong selection (e.g., complete sweep), hence we compared populations that exhibited the largest differences in allele frequencies. However, to detect both types of selection (strong and weak), multiple detection methodologies need to be utilised.

### 4.4 An outlier SNP associated with a candidate gene for WSC accumulation

Of the 33 SNPs common to more than one analysis method, one SNP was identified as located within a gene of biological significance, indicating a potential functional association. A white clover homologue of early responsive to dehydration (*ERD*) monosaccharide vacuole transporter, *ERD6-like 4* (At1g19450), was physically associated with SNP 16_32428574, detected in both the WNZLL and FNZSL pools. *ERD6*-like transporters are involved in energy-independent sugar efflux from the vacuole (Wei et al., 2014) and are induced by dehydration and cold treatment (Kiyosue et al., 1998). Endler et al. (2006) identified *ERD6-like 4* as being highly homologous to the sugar beet (*Beta vulgaris*) hexose transporter *U43629* (Chiou and Bush, 1996). This sugar transporter has been hypothesised to catalyse facilitated diffusion of glucose across the vacuole membrane (Chiou and Bush, 1996). In plants it is uncommon for monosaccharide transporters to function as facilitators (Büttner, 2007; Taiz and Zeiger, 2010). However, in the last decade Sugars Will Eventually be Exported Transporters (*SWEET*) proteins were discovered (Chen et al., 2010), which function as energy-independent uniporters of sucrose and glucose at the plasma membrane (Chen et al., 2010; Chen et al., 2012), and of fructose across the tonoplast membrane (Chardon et al., 2013). This evidence suggests a role for *ERD6-like 4* in WSC accumulation. It is possible that *ERD6* homologs are involved in movement of sugars out of the vacuole during circumstances where carbohydrate reallocation is important (Büttner, 2007). Although there is compelling evidence to suggest that *ERD6-like 4* plays a role in WSC accumulation, possibly in the form of osmotic adjustment, further experiments need to be done to test an hypothesis that *ERD6-like 4* protein expression leads to differences in WSC content in white clover leaves.

### 4.5 Putative candidate genes identified from GWAS

GWAS failed to identify SNPs significantly associated with variation in leaf WSC levels, after accounting for population structure. The false discovery rate applied was controlled by Bonferroni’s multiple testing correction method, which has been suggested to be too stringent (Wang et al., 2005; Hirschhorn and Daly, 2005) and may exclude real associations (Yang et al., 2010). Other studies have used a conservative *p*-value (*p* < 0.001) approach to reduce Type I error, whereby SNPs that are the highest ranking and have -log_10_(*p*-value) ≥ 3.0 are reported (Kang et al., 2015; Sakiroglu and Brummer, 2017; Biazzi et al., 2017). Applying this approach in the current study, ten SNPs in the SSS plot exceeded the log_10_(*p*-values) 3.0 threshold and two SNPs in the WSC plot exceeded the log_10_(*p*-values) 3.0 threshold. Seven candidate genes in close proximity to these highly ranked SNPs were investigated further for their potential association with genes.

SNP 5_47903593 is associated with *glgC*, encoding the small subunit of ADP-glucose pyrophosphorylase, which is involved in starch biosynthesis and in turn may affect glucose and sucrose concentrations (Burgess et al., 1997). Zhang et al. (2015) also found the large subunit of *glgC* (Glucose-1-phosphate adenylyltransferase) to be associated with drought resistance in *Medicago sativa*. This suggests that *glgC* may also play a role in osmotic adjustment or osmoprotection, affecting the capacity of cells to accumulate solutes and lowering water potential during periods of osmotic stress. Low molecular weight sugars are often involved in osmotic adjustment which adds to the confidence of associating variation in *glgC* with WSC accumulation in white clover.

Two SNPs (3_51874121 and 3_51874123) occur in genes associated with regulation of transcription, while SNP 11_7689073 is associated with translation initiation. Another SNP (9_23070656) was associated with the *UPL6* gene, which is involved in protein post-translational modification, mediating the addition of ubiquitin groups to target proteins for subsequent proteasomal degradation. Increased protein degradation due to environmental stress has been observed in plants as a way to mobilise nitrogen or eliminate damaged proteins (Eckert et al., 2010a). This may affect the ratio of protein to sugar concentrations in the cells as other studies have found evidence of induced protein turnover (enhancement of ubiquitin ligase) in response to water deficit and cold stress (Kiyosue et al., 1996; Abernethy and McManus, 1999; Schulze et al., 2003; Kim and Kim, 2013; Patel et al., 2015). A sixth SNP (1_2338028) occurred in the exon of *VPS35B*, which plays a role in vesicular protein sorting (Yamazaki et al., 2008) and is involved in plant growth and leaf senescence. Similarly, SNP 8_27636527 was associated with *COG8* which is involved in protein transport. Finally, SNP 1_102715 is associated with the chlorophyll a-b binding protein 7 gene, which functions as a light receptor that captures and delivers excitation energy to photosystem I. Expression of these genes will need to be investigated further to identify their role in WSC accumulation in white clover.

Inostroza et al. (2018) investigated WSC in white clover and identified four candidate genes associated with stolon WSC degradation rate, including a prolyl 4-hydroxylase alpha-like protein, a putative RING-finger E3 ubiquitin ligase, plant invertase/pectin methylesterase inhibitor and peptide/nitrate transporter. None of these genes were in common with the candidates identified in the current study, but there are important differences between the two investigations. Firstly, Inostroza et al. (2018) examined the stolon, typically a carbon sink, whereas leaves, as investigated in the current study, are carbon sources and the control of WSC in both tissues are likely to be under different mechanisms (Ballicora et al., 2004). Secondly, Inostroza et al. (2018) examined degradation of WSC whereas in the current study WSC accumulation was the focus. Finally, Inostroza et al. (2018) completed GWAS using a dataset of a greater SNP density (16.6 SNPs per Mbp) than the current study (6 SNPs per Mbp), therefore all genes important for WSC accumulation may not have been detected. The genetic control of foliar WSC accumulation requires further elucidation and should be investigated further.

## 5 Conclusions

The genetic control of foliar WSC accumulation in white clover was examined for the first time, by examining genetic changes in five breeding pools subject to divergent selection. Breeding for divergent foliar WSC was successful in all pools, with significant differences in WSC between low and high WSC populations achieved at the conclusion of the recurrent selection programmes, and these differences were not attributable to changes in leaf area. Outlier analyses identified GBS SNP markers that differentiate low and high WSC populations and, from these and from GWAS, two strong candidate genes were identified: *ERD6-like 4* and *glgC*. SNPs associated with a range of other candidates were also identified, which are involved in numerous aspects of plant development, membrane transport, post-translational processing, cell division and pathogen response. The clear phenotypic separation of the high and low WSC populations provides a robust platform for further investigation of foliar WSC accumulation in white clover, using transcriptomics and proteomics.

## Code availability

The code can be found publicly available on GitHub. https://github.com/SofiePearson/White_Clover_WSC_Outlier_Detection_GWAS.

## Data availability statement

The original contributions presented in the study are publicly available. The DNA sequence data have been deposited in the NCBI SRA database with links from BioProject accession number PRJNA915069. The phenotype data used in the study are provided in **Supplementary Tables 13** and **14**.

## Author contributions

SP contributed to experimental design, analysed phenotypic data, conducted laboratory work, undertook SNP filtering and genetic analyses, and contributed to the writing of the manuscript. MF and AG conceived and designed the study and contributed to the writing of the manuscript. CM developed the experimental design and PPM and PM completed statistical analyses of data. AL and SH completed laboratory work supporting GBS, and RJ performed raw sequence processing and SNP calling. JF bred the white clover populations used in the experiment. JT and PL contributed to writing the final version of the manuscript. All authors contributed to the article and approved the submitted version.
