# Supplementary material for: Outlier analyses and genome-wide association study identify glgC and ERD6-like 4 as candidate genes for foliar water-soluble carbohydrate accumulation in Trifolium repens
Source: Front Plant Sci. 2023 Jan 9;13:1095359. doi: 10.3389/fpls.2022.1095359 (PMC9868827; doi:10.3389/fpls.2022.1095359)
Supplement: Supplementary file 1 [file DataSheet_1.zip › Figures S1 -S12.docx]

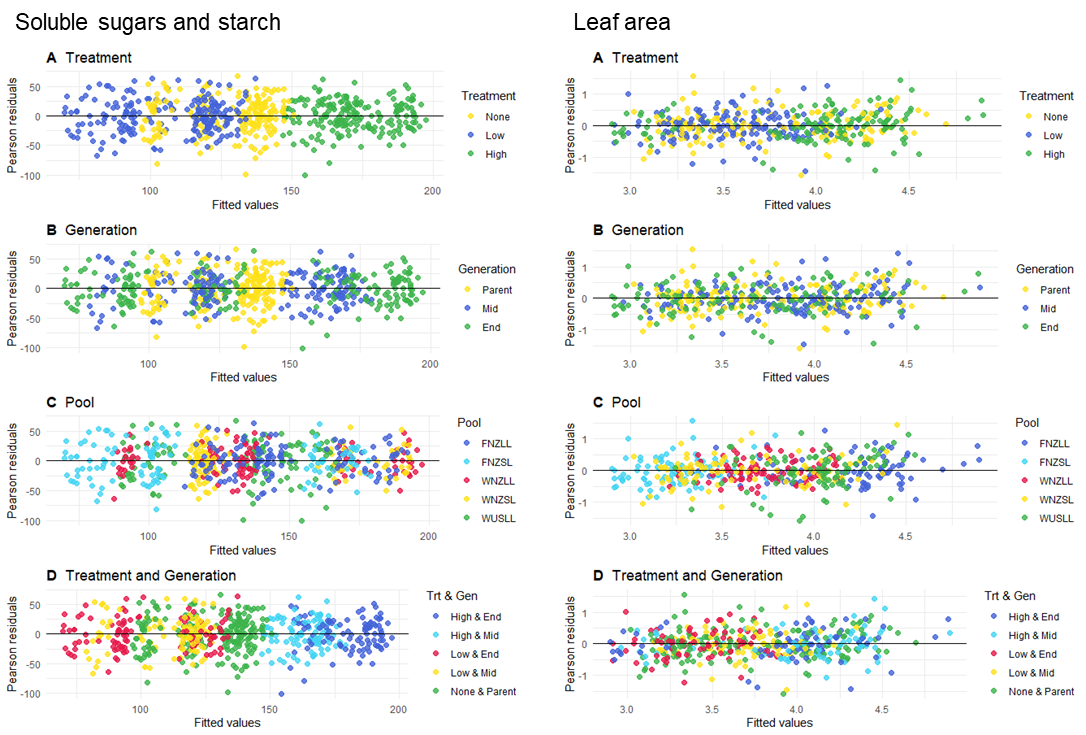


**Supplementary Figure 1** Residual plots for soluble sugars and starch (SSS) values by treatments (left) and square root leaf area values by treatment (right). Individuals were categorised into: (**A**) Treatment = None (i.e., Parent), Low or High, (**B**) Generation = Parent, Mid or End, (**C**) Pool = FNZLL, FNZSL, WNZLL, WNZSL and WUSLL, (**D**) Treatment and Generation combination = combination of treatment and generation. *Note*: Low = low water-soluble carbohydrate (WSC), High = high WSC, Trt = treatment, and Gen = generation.


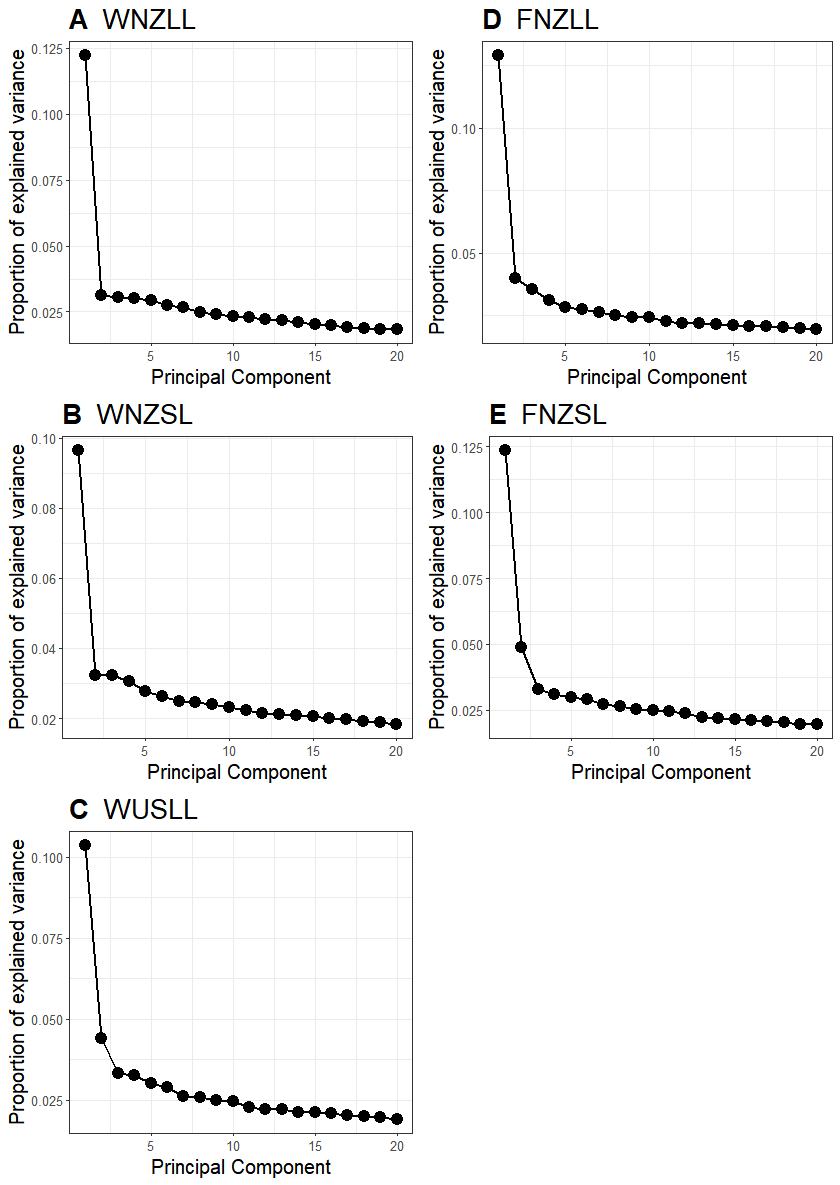


**Supplementary Figure 2** Scree plots for each pool determined in PCAdapt analysis. Proportion of explained variance is displayed on the *y*-axis with *K_PC_* values (number of principal components) from 1 to 20 displayed on the *x*-axis.


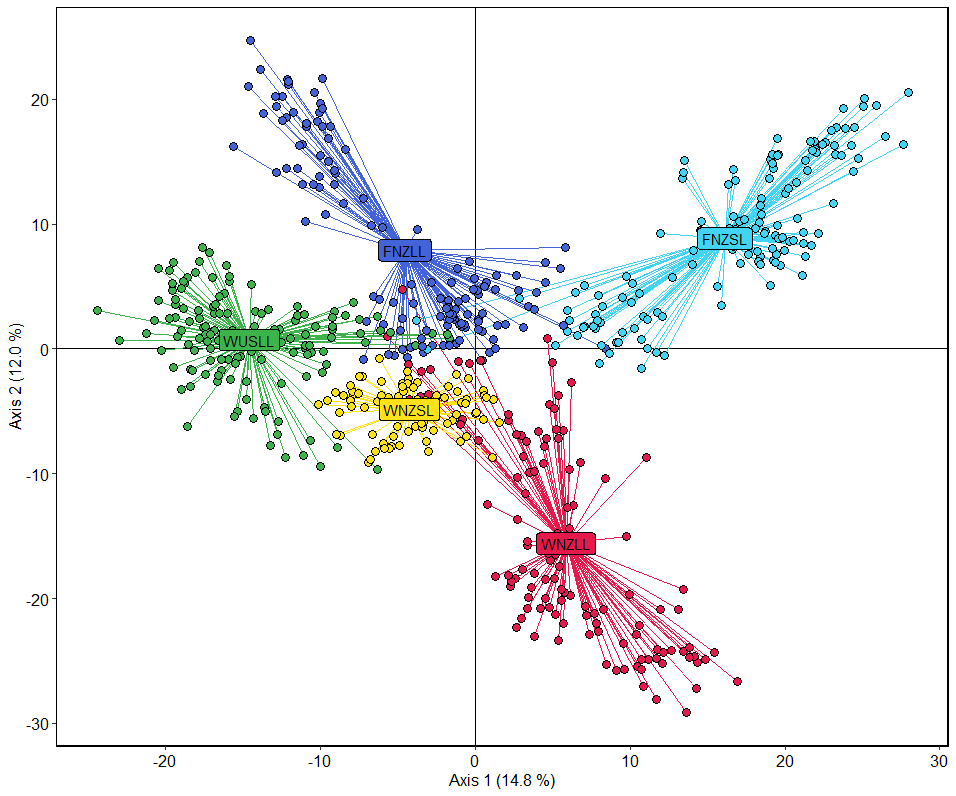


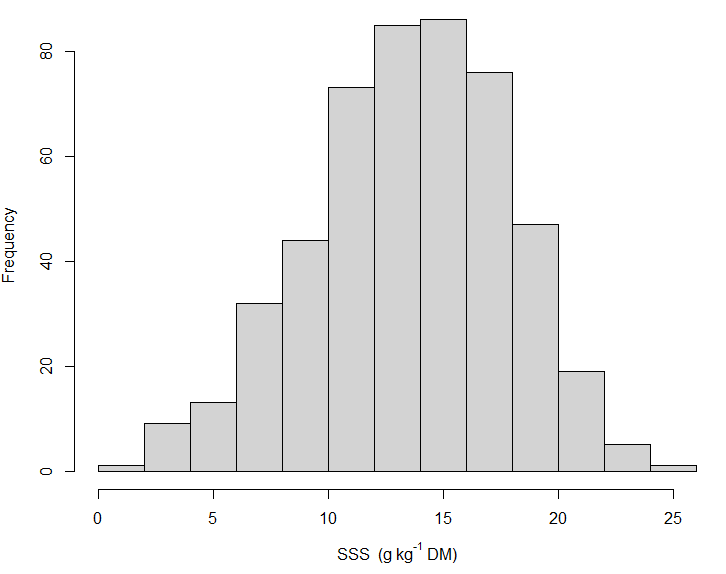


**Supplementary Figure 3** Summary information for individuals used in genome-wide association analysis. Principal component analysis of 605 samples and 5757 SNPs used for GWAS analysis (top) and distribution of SSS data (bottom).


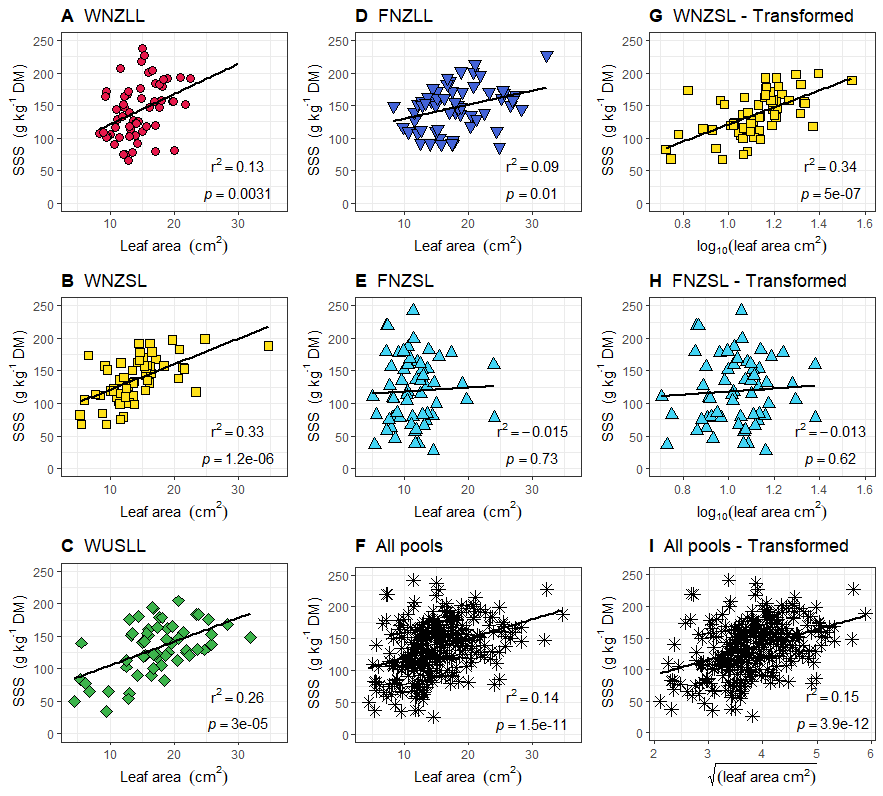


**Supplementary Figure 4** Correlation scatterplots between soluble sugars and starch (SSS) and leaf area for each pool and combined datasets. Raw data was used in plots A to F and log and square root transformed leaf area is presented in plots G to I. (**A**) SSS and leaf area correlation for WNZLL pool, *n* = 60. (**B**) SSS and leaf area correlation for WNZSL pool, *n* = 60. (**C**) SSS and leaf area correlation for WUSLL pool, *n* = 57. (**D**) SSS and leaf area correlation for FNZLL pool, *n* = 60. (**E**) SSS and leaf area correlation for FNZSL pool, *n* = 60. (**F**) SSS and leaf area for all data, *n* = 297. (**G**) SSS and log_10_ leaf area correlation for WNZSL pool, *n* = 60. (**H**) SSS and log_10_ leaf area correlation for FNZSL pool, *n* = 60. (**I**) SSS and square root leaf area for all data, *n* = 297. Adjusted r^2^ values and *p*-values for lines of best fit are shown.


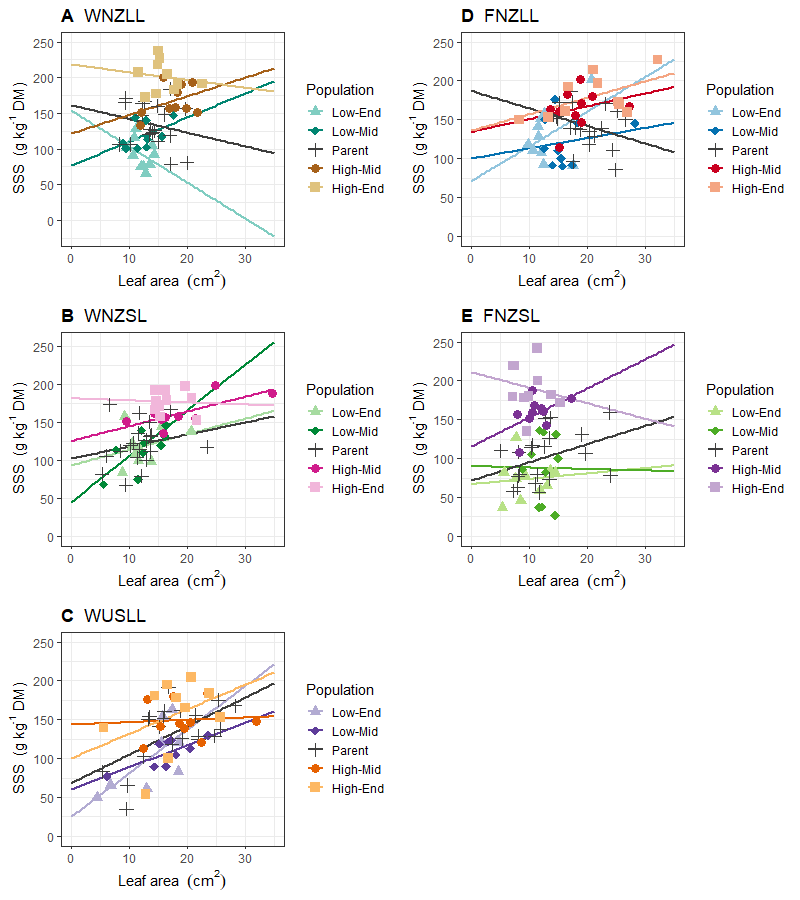


**Supplementary Figure 5** Regression scatterplots for soluble sugars and starch (SSS) and leaf area broken down to populations for each pool. (**A**) WNZLL pool, *n* = 60, (**B**) WNZSL pool, *n* = 60, (**C**) WUSLL pool, *n* = 57, (**D**) FNZLL pool, *n* = 60, (**E**) FNZSL pool, *n* = 60. Line of best fit equations for each population can be found in **Supplementary Table 3**. Overall adjusted r^2^ values and *p*-values for lines of best fit for each pool are shown in **Supplementary Figure 4**.

**Supplementary Figure 6** Relationship between the number of filtered SNPs per pseudomolecule and pseudomolecule size (Mbp) using samples from all pools (*n* = 1,113). SNPs were filtered to include only biallelic SNPs, a minimum and maximum read depth range of 5 to 150, maximum missing data to 20% per SNP and including SNPs with a minor allele frequency of ≥ 0.03. Square data points represent the Tr_To_ pseudomolecule and the circular data points represent the Tr_Tp_ pseudomolecule. Line of best fit and coefficient of determination (square of the Pearson correlation, r^2^) are presented.


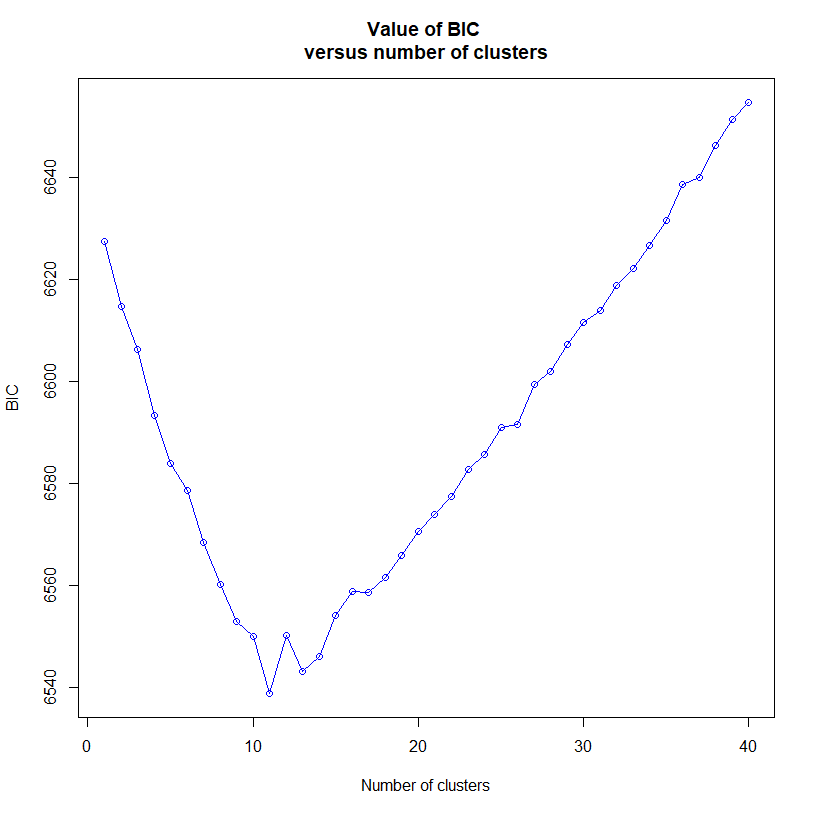


**Supplementary Figure 7** Selection of the optimal number of clusters for discriminant analysis of principal components (DAPC) using *K*-means algorithm and the lowest Bayesian information criterion (BIC). The graph shows a clear decrease of BIC until *K* = 11 clusters as the most likely value of *K*, after which BIC increases.

**Supplementary Figure 8** Cross-validation results from discriminant analysis of principal components (DAPC) for *K* = 11. Proportion of successful assignment of the validation set (10% of the data) is presented on the *y*-axis and the number of principal components (PCs) is presented on the *x*-axis (every second PC is represented). Mean values from 100 replicate runs for each PC are presented for mean successful assignment (MSA) as blue squares, and root mean square error (RMSE) as green circles. RMSE and MSA values plateau at six PCs as indicated by the black points.


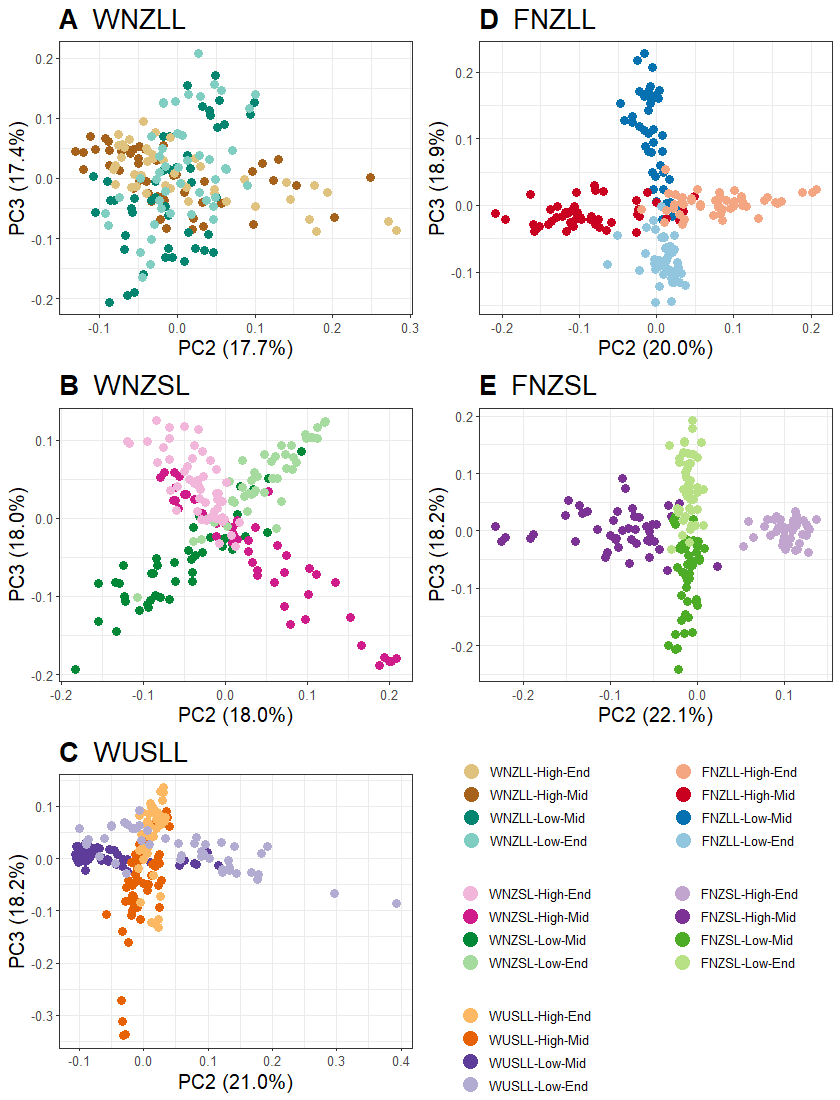


**Supplementary Figure 9** Score plots from PCAdapt analysis for each pool with principal component (PC) 2 and PC 3 displayed. Each dot represents a single individual and the colour corresponds to individuals from the same population. Each pool has four populations as the Parent populations was excluded from the analysis. Population information is displayed in the key in the bottom right corner.


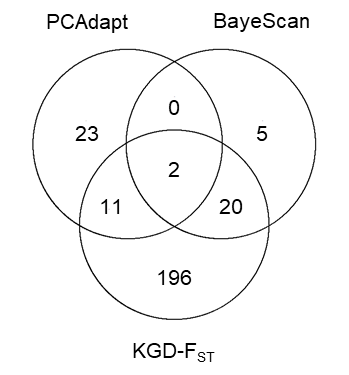


**Supplementary Figure 10** Venn diagram of the overlap between loci detected by PCAdapt, BayeScan and KGD-F_ST_. PCAdapt and BayeScan false discovery threshold α = 0.05 was used, and SNPs in common between two or more pools at an F_ST_ > 0.3 was used for KGD-F_ST_ false discovery criteria. The 33 SNPs found as outliers in two or more analyses are considered to be strong candidates for selection.


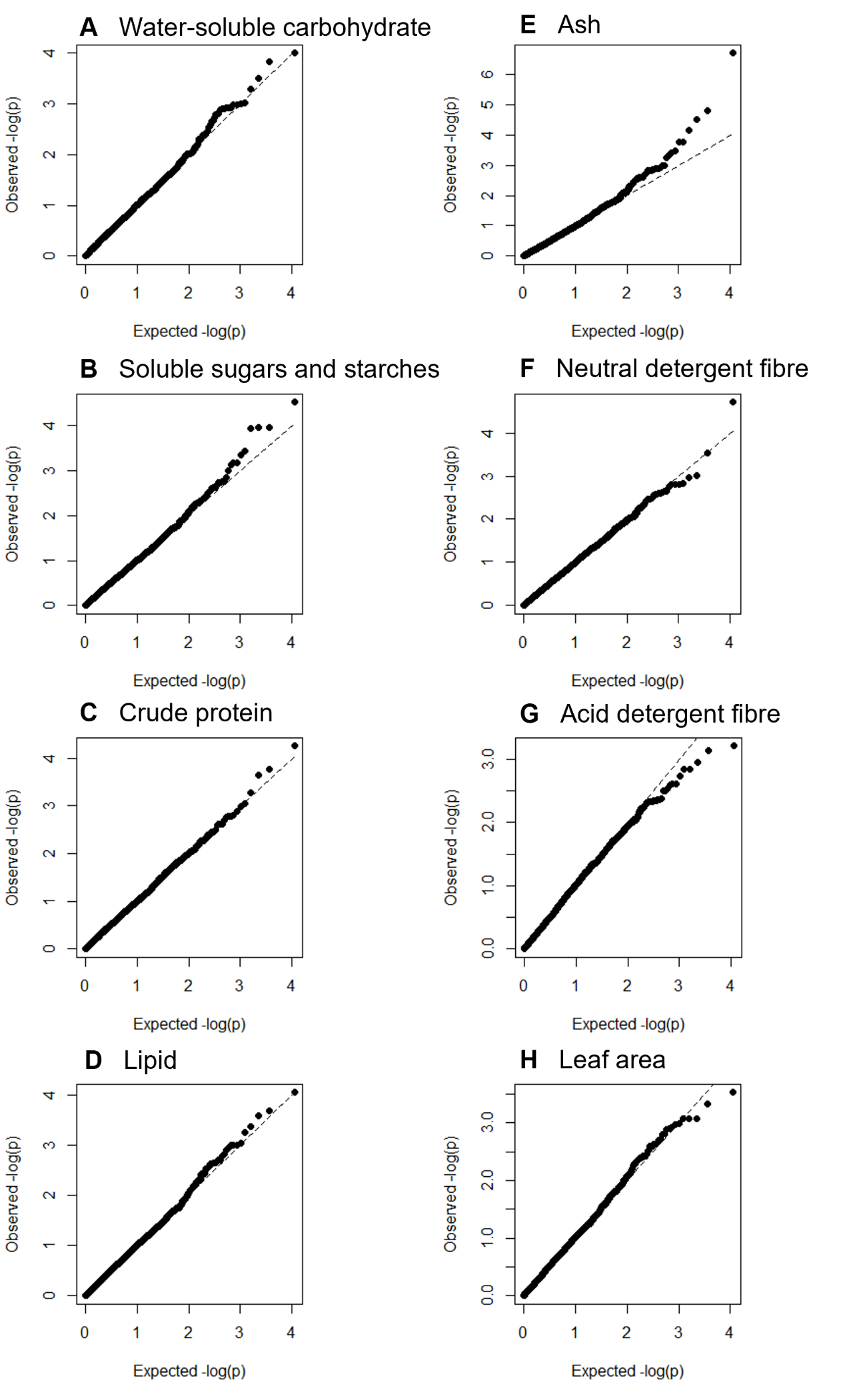


**Supplementary Figure 11** Quantile-Quantile (Q-Q) plots of expected *p*-values on *x*-axis and observed *p*-values on *y*-axis for each SNP in eight phenotypic traits investigated in the genome-wide association study (GWAS) (**Figure 6** and **Supplementary Figure 12**). Most *p*-values are similar to the expected diagonal in the Q-Q plots, which indicates the appropriateness of the GWAS model.


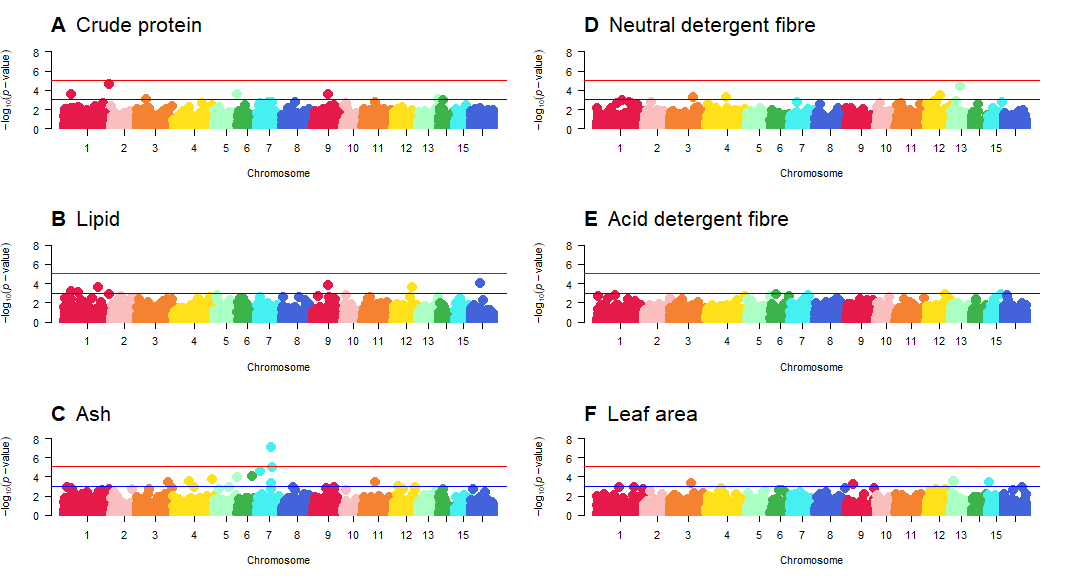


**Supplementary Figure 12** Manhattan plots from the genome-wide association study (GWAS) of six phenotypic traits using 5,757 SNP markers and 605 individuals. -log_10_(*p*-values) are plotted against physical map position of SNPs with subgenomes of corresponding chromosomes (i.e., pseudomolecules) similarly coloured (Tr_To_ 1 – 8 and Tr_Tp_ 9 – 16). Significant loci lie above the false discovery rate thresholds as denoted by the red (α = 0.01) and blue (-log_10_(*p*-value) > 3) solid lines. Quantile-Quantile plots for each trait are presented in **Supplementary Figure 11**.

**Supplementary methods**

**Bisbenzimidazole Hoechst 33258 DNA quantification**

DNA was diluted 1:3 in TE buffer (i.e., 15 μL DNA added to 30 μL TE in 96-well MultiMax (vwr.com) plates. The solutions for Hoechst quantification are as follows. TNE (2 M NaCl) was prepared according to Rago *et al.* (1990). Sufficient Hoechst dye reaction mixture was made for quantification of 96 DNA samples plus 8 standards in triplicate by adding 17.8 mL TNE and 200 μL Hoechst dye stock (Thermo Fisher Scientific Inc.) into a 50 mL Falcon tube. The Phage λ DNA (Boehringer Mannheim, Indianapolis) standards were diluted to a range of concentrations between 5 and 50 ng μL^-1^ to create a linear set of standards according to the following scheme:

| Final concentration (ng µL^-1^): | 0 | 5 | 10 | 15 | 20 | 25 | 30 | 35 |
| --- | --- | --- | --- | --- | --- | --- | --- | --- |
| 100 ng µL^-1^ λ DNA: | 0 | 50 | 100 | 150 | 200 | 250 | 300 | 350 |
| _s_H_2_O: | 1000 | 950 | 900 | 850 | 8000 | 750 | 700 | 650 |

A 45 μL aliquot of Hoechst dye reaction mixture was added to each well of a black 384-well Black Assay Plate (4titude, Surrey UK). Each DNA sample was assayed in triplicate, with 5 μL of diluted DNA added to the Hoechst dye reaction mixture and 5 μL of undiluted standard added to the Hoechst dye reaction mixture and then the 384-well plate was quantified by measuring fluorescence using a Synergy HTX Multi-Mode Microplate Reader (BioTek, VT, USA). The fluorescence values converted to ng μL^-1^ based on the Phage λ DNA standard curve. DNA samples were stored at -20°C until needed for GBS library construction.

**GBS library preparation**

For each individual, genomic DNA concentration was normalised to 100 ng μL^-1^ for library construction and was added to a dried down 96-well adapter plate using a Nanodrop II (BioNex Solutions, CA, USA). Each well of the plate had a unique barcoded adapter to enable subsequent sample identification. The plate was sealed with an Air-O-Seal sheet (4Titude, Surrey, EU), spun down briefly and then dried down using the Savant SPD111V SpeedVac Concentrator (ThermoFisher, MA, USA). A digestion for one reaction was performed in a 20 μL volume containing 16 μL nuclease-free water, 2 μL CutSmart Buffer (New England Biolabs Inc. (NEB), Ipswich, MA, USA, No. B7204S), 1 μL 20 U μL^-1^ *PstI* (NEB, Ipswich, MA, USA, No. R0140S), and 1 μL 20 U μL^-1^ *MspI* (NEB, Ipswich, MA, USA, No. R0106S) which was added into the adapter + DNA plate. Enzymatic digestion was carried out using a Kyratech Thermocycler (ThermoFisher, MA, USA) and included an incubation period of 2 hours at 37°C and 30 minutes at 65°C. Ligation of adapters onto fragmented DNA was performed for each digested sample by adding a 30 μL reaction solution containing 5 μL 10X Ligase buffer (NEB, Ipswich, MA, USA, No. B0202S), 2 μL T4 DNA Ligase (NEB, Ipswich, MA, USA, No. M0202L) and 23 μL nuclease free water. Ligation was carried out using a Kyratech Thermo cycler (ThermoFisher, MA, USA) and included ligation at 22°C for one hour and incubation at 65°C for 20 minutes. A 5 μL aliquot of each digested/ligated sample was then pooled together into a single well of a PCR strip tube using a Nanodrop II (BioNex Solutions, CA, USA). The pooled samples were then transferred from the PCR strip tube into a single 5 mL Eppendorf tube (Eppendorf) with 2.5 mL CP buffer (Omega Bio-Tek, GA, USA). Libraries were purified with an E.Z.N.A. Cycle Pure Kit (Omega Bio-Tek, GA USA, No. D6492-02), per kit instructions and then eluted in 50 μL elution buffer (Omega Bio-Tek, GA, USA). To increase the amount of GBS library DNA for size selection prior to sequencing, six parallel polymerase chain reaction (PCR) amplifications were completed for each library. Restriction fragments from each library were amplified in six separate reactions, each in a 50 μL volume containing 4 μL pooled DNA fragments, 25 μL 2X Taq Master Mix (NEB, Ipswich, MA, USA, No. M0270L), 2 μL PCR primer Mix (12.5 pmol μL^-1^ each primer as described in Poland et al. (2012)), and 19 μL nuclease free water. Amplification by PCR was carried out using a Kyratech Thermo cycler (ThermoFisher, MA, USA) and included an initial denaturation at 72°C for 5 minutes and 98°C for 30 seconds; then 18 cycles at 98°C for 10 seconds, 65°C for 30 seconds and 72°C for 30 seconds; followed by a final extension at 72°C for 5 minutes. For each PCR product, all six parallel samples were pooled and purified with an E.Z.N.A. Cycle Pure Kit (Omega Bio-Tek, GA USA, No. D6492-02) per kit instructions and eluted in 35 μL elution buffer. Libraries were assessed for quantity and quality on a nanodrop Spectrophotometer (Nanodrop Technologies, Montchanin, USA) using a 1.5 μL aliquot of library. A 2 μL aliquot of the library was reserved for analysis prior to size selection. The fragment size selection step was performed on a 30 μL aliquot of the library which was combined with 10 μL of size ladder L (Pippin reagents kit No.: CDF2010) and then DNA fragments of 193 – 313 bp size range were selected on a Pippin Prep (Sage Science, MA, USA). A 2 μL aliquot of the size-selected library was reserved for analysis. The pre-size selection aliquot was diluted 1:5 (6-fold dilution) and the post-size selection sample was diluted 1:4 (5-fold dilution) with water. An Agilent 2100 Bioanalyzer (Agilent Technologies, CA, USA) was used to assess the pre- and post-size selection samples and quality of each library.
